# Single cell profiling reveals malignant states and immune landscapes in PCNSL and systemic DLBCL

**DOI:** 10.1016/j.isci.2026.115563

**Published:** 2026-04-02

**Authors:** Fuqiang Cai, Xiaofang Wang, Shunjie Zhang, Yumiao Li, Shenghan Wang, Jijun Zhu, Pan Li, Zhongting Huang, Weixin Liu, Zhijian Song, Chang Tian, Yan Li, Guiliang Han, Junfang Chen, Fei Ling, Youchao Jia

**Affiliations:** 1 School of Biology and Biological Engineering, South China University of Technology, Guangzhou, China; 2 Department of Medical Oncology, Affiliated Hospital of Hebei University, Hebei Key Laboratory of Cancer Radiotherapy and Chemotherapy, Hebei, China; 3 Center for Intelligent Medicine, Greater Bay Area Institute of Precision Medicine (Guangzhou), School of Life Sciences, Fudan University, Shanghai, China; 4 Center for Evolutionary Biology, School of Life Sciences, Fudan University, Shanghai, China; 5 Department of Haematology, Hebei General Hospital, Shijiazhuang, Hebei, China

**Keywords:** health sciences, medicine, oncology

## Abstract

Primary central nervous system lymphoma (PCNSL) is a rare, aggressive subtype of diffuse large B-cell lymphoma (DLBCL) with distinct biology. We present the first integrative single-cell transcriptomic atlas comparing PCNSL and systemic DLBCL (sDLBCL), profiling 171,322 cells from 31 patients. We identified five malignant B-cell subtypes with discrete differentiation trajectories. A CNS-enriched progenitor-like B0 subtype with MYC/VEGFA activation predominated in PCNSL and was linked to poor prognosis. PCNSL also exhibited a highly immunosuppressive microenvironment with exhausted cytotoxic T cells, M2-like macrophages, and elevated PD-L1, TIGIT, and BTLA. In contrast, sDLBCL showed more inflammatory signatures. Co-expression network and pharmacogenomic modeling revealed subtype-specific transcriptional modules associated with resistance and outcome. Modules ME7/ME15 were enriched in PCNSL, while ME17 marked drug resistance in sDLBCL. These findings establish PCNSL as a transcriptionally and immunologically distinct entity and provide a rationale for targeted immunotherapy.

## Highlights

• Single-cell atlas of PCNSL vs. sDLBCL reveals five malignant B-cell subtypes
• CNS-enriched progenitor-like B0 subtype drives PCNSL and predicts poor prognosis
• PCNSL shows immunosuppressive T cell and macrophage states with checkpoint elevation
• Co-expression modules uncover subtype-specific resistance and therapeutic targets

## Graphical abstract

**Figure:**
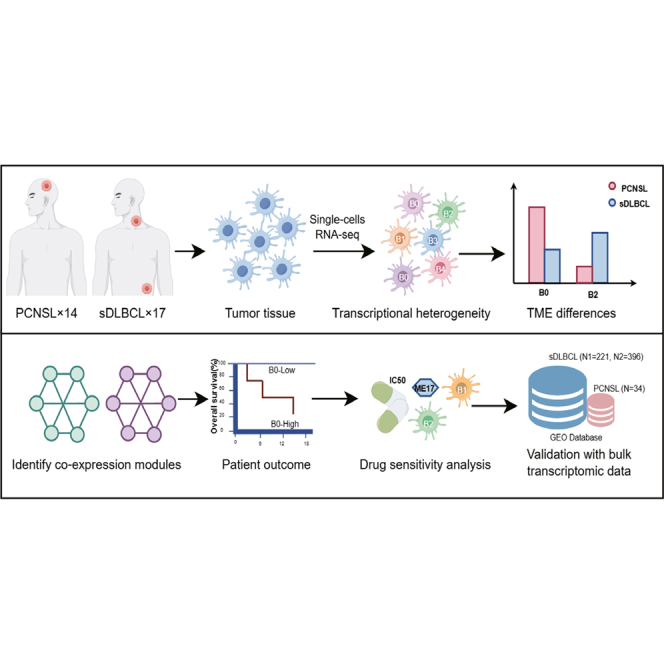

## Introduction

Diffuse large B-cell lymphoma (DLBCL) is the most common subtype of non-Hodgkin lymphoma, noted for its clinical aggressiveness and molecular heterogeneity. Despite advances in immunochemotherapy, outcomes vary widely across patients, driven by a complex interplay of tumor-intrinsic factors, host genetics, treatment-related variables, and social determinants of health. Epidemiologic studies, including the Lymphoma Epidemiology of Outcomes study, have highlighted disparities across age, ancestry, and socioeconomic strata, reflecting the complex interplay between biology and systemic determinants.^1^ At the molecular level, DLBCL comprises genetically distinct subtypes defined by cell-of-origin (ABC and GCB), recurrent mutations (e.g., *MYD88*, *CD79B*, and *EZH2*), copy number alterations, and structural variants, each associated with unique oncogenic pathways and therapeutic vulnerabilities.^2^^,^^3^ When DLBCL arises in the central nervous system (CNS), either as a primary malignancy (PCNSL) or by secondary spread, it presents with rapid progression, limited therapeutic response, and poor prognosis.^4^^,^^5^ PCNSL exhibits distinct immune microenvironment subtypes, immune-rich, intermediate, and poor, that are associated with differential signaling activation and immune evasion mechanisms.^6^ Recent studies have further refined prognostic stratification by identifying specific molecular markers; for instance, the reciprocal expression of immune response genes such as *CXCR3* and *IFI44L*, as well as distinct microRNA signatures associated with cancer immunity, have been linked to patient survival in PCNSL.^7^^,^^8^^,^^9^ Despite increasing clinical awareness, the molecular and cellular landscape of CNS-involved DLBCL remains poorly characterized, limiting the development of effective precision therapies.

Single-cell RNA sequencing (scRNA-seq) has transformed our ability to resolve intratumoral heterogeneity, lineage plasticity, and immune architecture across hematologic malignancies.^10^^,^^11^ Studies have begun to reveal the transcriptional complexity of systemic DLBCL at single-cell resolution,^12^^,^^13^ and functionally defined lymphoma microenvironment classes have been linked to prognosis and drug response.^14^ In PCNSL, frequent *MYD88* L265P and CD79B mutations activate BCR and NF-κB signaling, accompanied by epigenetic dysregulation, aberrant somatic hypermutation, and deletions of immune-regulatory loci such as *HLA-D* and *CDKN2A*.^15^^,^^16^ These findings suggest that PCNSL is molecularly distinct from systemic DLBCL. Yet, no study has systematically compared PCNSL and systemic DLBCL at single-cell resolution. The CNS poses a unique immunologic environment shaped by the blood-brain barrier, restricted lymphatic drainage, and limited antigen presentation, which may selectively shape malignant evolution and immune evasion.^17^^,^^18^ Bulk profiling approaches fail to capture such tissue-specific adaptation and obscure the diversity of malignant and immune compartments.^19^

To address this gap, we constructed a high-resolution single-cell atlas of PCNSL and systemic DLBCL to define malignant B-cell states, immune cell types, and transcriptional modules across anatomical contexts. We hypothesized that CNS-resident DLBCL contains distinct malignant populations driven by the immunoprivileged microenvironment, with enhanced proliferative and invasive potential contributing to poor outcomes. We also proposed that identifying subtype-specific gene programs could inform prognosis and therapeutic response.

We analyzed 171,322 single cells from 14 PCNSL and 17 systemic DLBCL samples using an integrative framework combining copy number inference, differentiation trajectory analysis, cell-cell communication modeling, co-expression network analysis (hdWGCNA), and transcriptome-based drug response prediction. This enabled fine-grained mapping of malignant and immune architecture across compartments. We found that PCNSL is dominated by a proliferative B0 subtype with MYC and VEGFA activity, while systemic DLBCL is enriched for an immunoregulatory B2 subtype linked to drug resistance and poor prognosis. PCNSL also exhibits profound T cell exhaustion and macrophage suppression, contrasting with the more cytotoxic, memory-rich immune environment of systemic DLBCL. Together, these findings provide a molecular framework for understanding tissue-specific adaptation and guiding stratified immunotherapy in aggressive B-cell lymphomas.

## Results

### Single-cell atlas of sDLBCL and PCNSL

**Figure 1:**
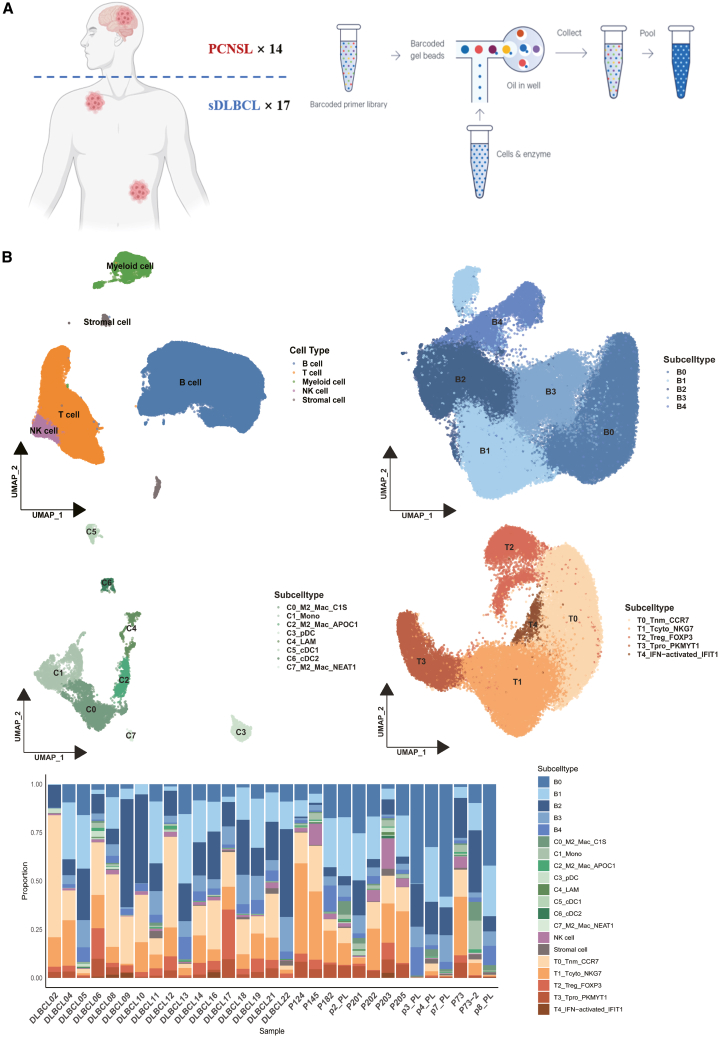
Single-cell landscape of PCNSL and sDLBCL (A) Schematic diagram of the sample cohort: scRNA-seq from 14 PCNSLs and 17 sDLBCLs were collected. The anatomical origin of the biopsy is indicated (brain, lymph node). (B) Above are four UMAP projections, displaying all cells (pan-microenvironment), B cell subtypes, T cell subtypes, and myeloid cell subtypes, with each cell type represented by a distinct color scheme. Below is a stacked plot of the cell subtype ratio for each sample.

Single-cell atlas of sDLBCL and PCNSL To comprehensively dissect the characteristics of aggressive B-cell lymphomas and elucidate immunomicroenvironmental heterogeneity and connectivity between PCNSL and sDLBCL, we constructed an integrated single-cell atlas based on 171,322 cells from 14 PCNSL and 17 sDLBCL samples. We defined the cell types of each cluster using typical marker genes, including B cells, T cells, NK cells, myeloid cells, and stromal cells (Figures 1B and S1A). Data on B cells, T cells, and myeloid cells were extracted, and a second round of cluster analysis was performed, identifying five B-cell subtypes, five T cell subtypes, and eight myeloid cell subtypes (Figure 1B; Table S6).

### Malignant B cell heterogeneity in sDLBCL and PCNSL

**Figure 2:**
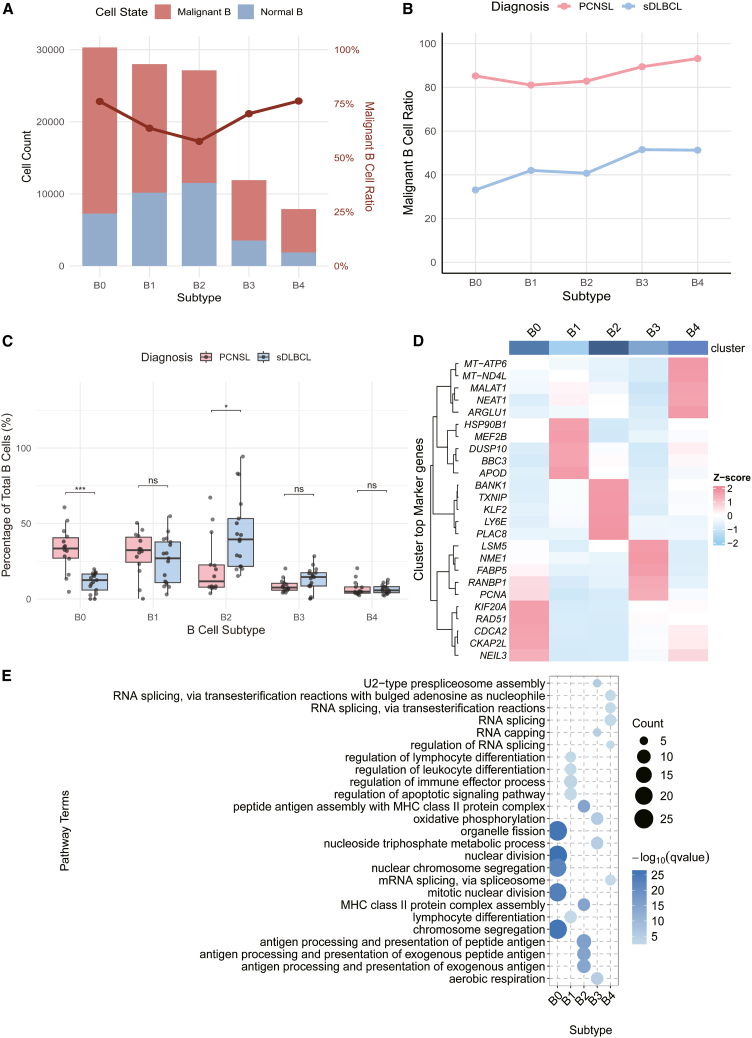
Differential B cell subset abundance and functional (A) The B-cell subtype composition between PCNSL and sDLBCL was compared, presenting a dual-axis visualization, where the left y axis (bar graph) shows the absolute counts of malignant B cells (red), and normal B cells (blue) in the different subtypes, while the right y axis (line graph) tracks the percentage of malignant B cells in each subtype. (B) Line graphs show the proportion of malignant B cells in each B cell subtype and their distribution in PCNSL (red) and sDLBCL (blue). (C) Boxplots compare B cell subtype proportions between PCNSL (red) and sDLBCL (blue), with Wilcox test indicated (∗*p* < 0.05, ∗∗*p* < 0.01, and ∗∗∗*p* < 0.001). (D) The heatmap shows the average expression levels of the top 5 DEGs in B cell subtypes. The gene expression levels were transformed by *Z* score. (E) The bubble chart shows the top 5 pathways of the GO pathway enrichment results of the top 50 DEGs of B cell subtypes. The bubble size indicates the number of enriched genes, and the depth of blue indicates -log10 (q value).

Malignant B-cell heterogeneity in sDLBCL and PCNSL using the inferCNV tool, we separately analyzed B cells from sDLBCL and PCNSL and rigorously classified them as malignant or normal based on HMM predictions. This approach identified 70,934 malignant B cells and 34,321 normal B cells. Malignant B-cell proportions across subclusters (B0-B4) showed distinct distribution patterns: B2 had the lowest malignant fraction (57.6%), while B0 and B4 had higher malignant fractions (76.1% and 76.3%) (Figure 2A). Comparative analysis demonstrated significantly elevated malignant proportions in all PCNSL subclusters compared to sDLBCL counterparts. Notably, the B0 subset exhibited the highest malignant proportion in PCNSL but the lowest in sDLBCL (Figure 2B). Complete malignant cell counts are provided in Table S3. Heatmap analysis revealed distinct gene expression patterns across B-cell subclusters: B0/B3/B4 showed minimal HLA and IG gene expression, B2 exhibited high expression of both gene families, and B1 had elevated IG gene expression (Figures S2A–S2D).

Comparative analysis revealed significant differences in B-cell subset distribution between sDLBCL and PCNSL. Specifically, B0 cells were markedly enriched in PCNSL compared to sDLBCL, whereas B2 cells were more prevalent in sDLBCL (∗*p* < 0.05 and ∗∗∗*p* < 0.001; Figure 2C). To validate the reliability of our single-cell-derived signatures, we applied CIBERSORTx deconvolution to bulk transcriptomes. Consistent with single-cell observations, the B0 subtype was significantly more abundant in PCNSL bulk samples, whereas B2 was enriched in sDLBCL (Figure S6A; Table S9). Survival analysis in these bulk cohorts confirmed the context-dependent clinical impact of the B0 subtype: A high fraction of B0 cells was significantly associated with shorter overall survival in PCNSL, whereas the opposite trend was observed in sDLBCL (Figures S6C and S7). To investigate the functional heterogeneity of B-cell subsets, we generated a heatmap displaying the top 5 DEGs for each subset (Figure 2D) and performed GO enrichment analysis on their top 50 DEGs (statistical significance was determined using Benjamini-Hochberg-adjusted *p* values <0.05) (Figure 2E; Table S7). GO analysis revealed distinct functional enrichment for each subset, indicating subtype-specific biological roles.

### Differentiation potential and developmental trajectory of malignant B-cell subtypes

**Figure 3:**
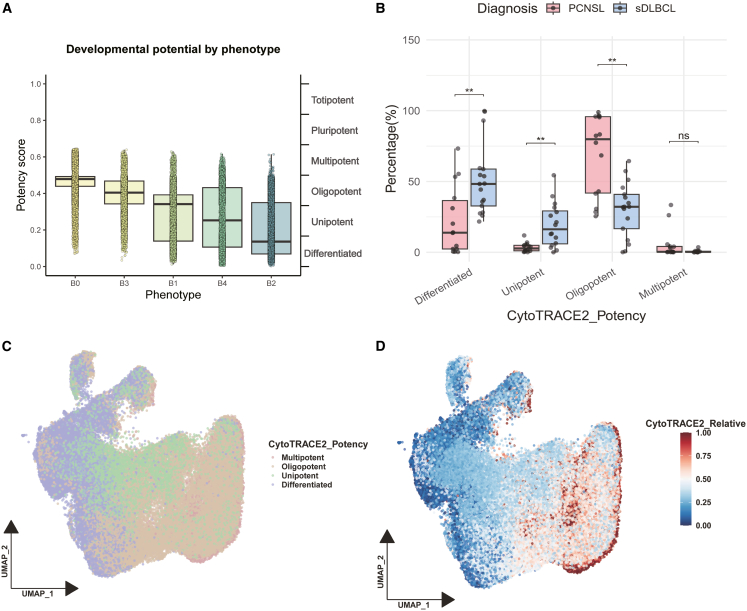
Differentiation capabilities and trajectories of B cell subtypes (A) The boxplot shows the potency score of each B cell subtype. (B) Comparison of the proportion of various potency types of B cells in PCNSL (red) and sDLBCL (blue), with Wilcox test indicated (∗*p* < 0.05, ∗∗*p* < 0.01, and ∗∗∗*p* < 0.001). (C) UMAP of CytoTRACE2 Potency Status of B Cells. (D) UMAP of CytoTRACE2 relative score for B cells. A higher score (red) means a stronger Potency.

To assess the differentiation capacity of malignant B-cell subsets, we applied CytoTRACE2, a computational tool for inferring cellular differentiation states. The results showed a differentiation hierarchy: B0 exhibited the highest differentiation potential, followed by B3, B1, B4, and B2 (Figure 3A). PCNSL samples had significantly more oligopotent cells, whereas sDLBCL had higher proportions of differentiated and unipotent cells (∗∗*p* < 0.01, Figure 3B). Trajectory analysis supported a differentiation path from progenitor-like B0 to terminally differentiated B2 (Figures 3C and 3D).

To orthogonally validate this hierarchy, Monocle-based trajectory inference was performed, which recapitulated the CytoTRACE2-inferred developmental ordering. In both PCNSL and sDLBCL, malignant B cells formed a continuous trajectory originating from the B0 subtype. Pseudotime-dependent gene modules in PCNSL revealed a transition from early cell-cycle progression to late metabolic and stress responses, whereas sDLBCL exhibited a more homogeneous trajectory dominated by continuous metabolic processes (Figure S3).

### Characterization of co-expression modules and malignant B cell subtype-specific patterns

**Figure 4:**
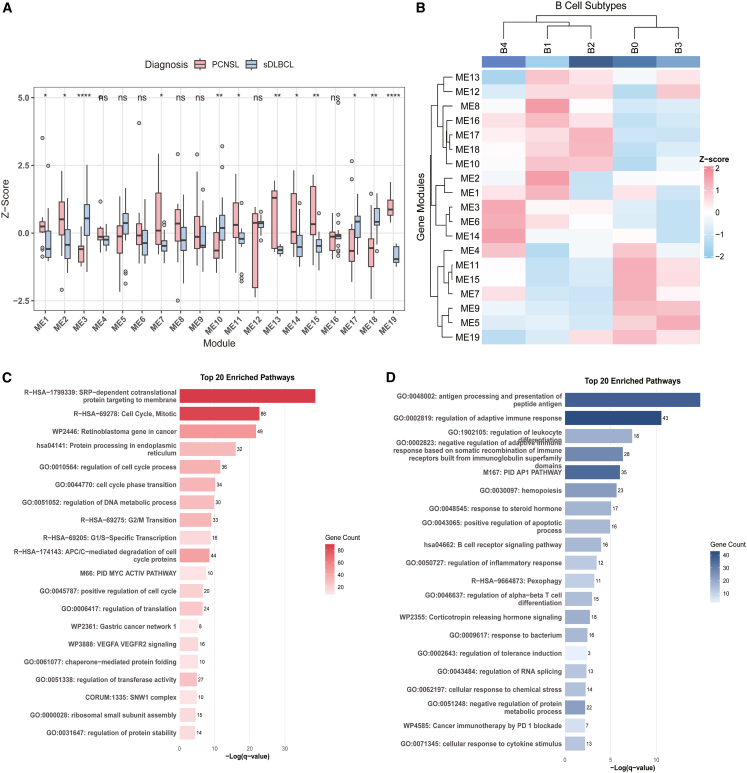
Specific patterns of gene co-expression modules in diseases (A) Comparison of the mean scores of each gene co-expression module in PCNSL (red) and sDLBCL (blue). The module scores were transformed by *Z* score, with Wilcox test indicated (∗*p* < 0.05, ∗∗*p* < 0.01, and ∗∗∗*p* < 0.001). (B) Heatmap shows the scores of the 19 gene co-expression modules in each B cell subtype; the module scores were transformed by *Z* score. (C) Metascape enrichment results of all hubgenes in the highly expressed module enriched in PCNSL (red). (D) Metascape enrichment results of all hubgenes in the highly expressed module enriched in sDLBCL (blue).

U22sing hdWGCNA on rigorously defined malignant B cells, we identified 19 distinct co-expression modules (ME1-ME19). The functional enrichment results of each module are shown in Figure S4D. By analyzing the top 25 hub genes from each module (the 25 hub genes can be found in Table S8), we compared module activity between sDLBCL and PCNSL. The analysis revealed that PCNSL showed significantly higher expression of ME1, ME2, ME7, ME11, ME13, ME14, ME15, and ME19, while sDLBCL exhibited elevated expression of ME3, ME10, ME17, and ME18 (adjusted *p* < 0.05, Figure 4A). Metascape analysis of hub genes from PCNSL-enriched modules demonstrated significant enrichment of cell cycle-related pathways, MYC22 activation, and VEGFA-VEGFR2 signaling, suggesting prominent roles in proliferation and angiogenesis (Figure 4C). To provide a molecular explanation for the poor prognosis associated with B0 in PCNSL, we evaluated specific oncogenic pathways. The B0 subtype exhibited significantly higher scores for MYC activation and VEGFA-VEGFR2 signaling compared to other subsets. Crucially, B0 cells derived from PCNSL displayed significantly higher activation of these pathways than those from sDLBCL, suggesting that the CNS microenvironment specifically exacerbates this aggressive, pro-angiogenic phenotype (Figures S5A–S5C). In contrast, sDLBCL-enriched modules were predominantly associated with antigen processing and presentation through MHC class II, along with the regulation of adaptive immune responses (Figure 4D), indicating distinct immune microenvironment interactions in these lymphomas.

Examination of module expression patterns across B-cell subtypes revealed that B0 and B3 cells showed high activity of ME5, ME7, ME9, ME11, ME15, and ME19. B1 and B2 cells displayed distinct expression of ME10, ME17, and ME18, while B4 cells specifically upregulated ME3, ME6, and ME14 (Figure 4B). These differential module activation patterns suggest subtype-specific functional specialization within the malignant B-cell populations.

### Analysis of T cells and myeloid differences in PCNSL and sDLBCL

**Figure 5:**
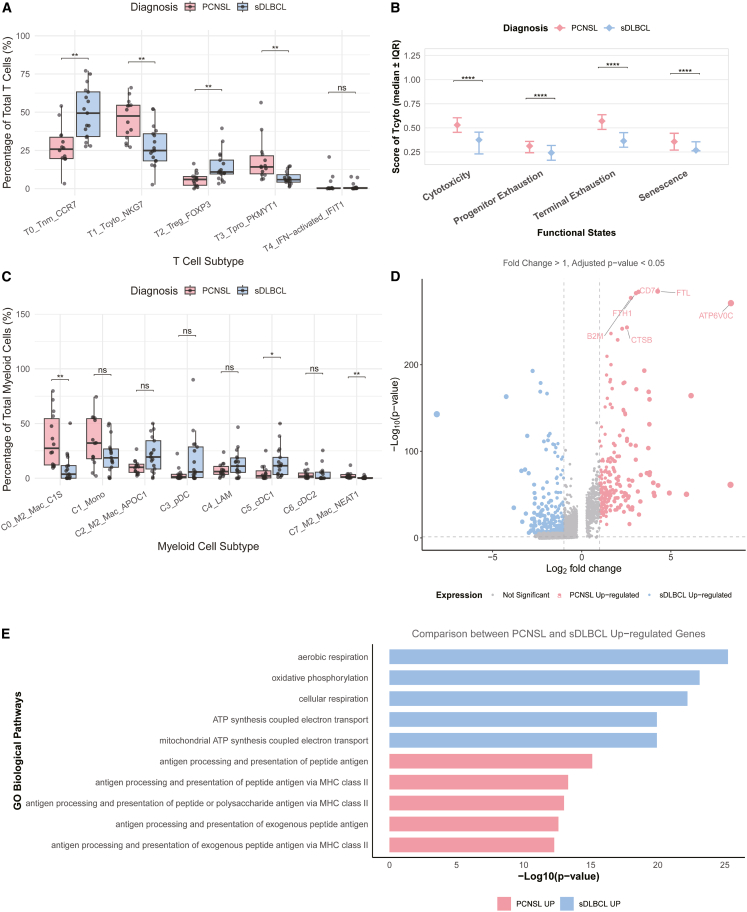
Heterogeneity of the tumor immune microenvironment (A) Boxplots compare T cell subtype proportions between PCNSL (red) and sDLBCL (blue), with wilcox test indicated (∗*p* < 0.05, ∗∗*p* < 0.01, and ∗∗∗*p* < 0.001). (B) Comparison of T cell status scores (including cytotoxicity, progenitor exhaustion, terminal exhaustion, and senescence) of Tcyto cells in PCNSL (red) and sDLBCL (blue), with wilcox test indicated (∗*p* < 0.05, ∗∗*p* < 0.01, and ∗∗∗*p* < 0.001). (C) Boxplots compare myeloid cell subtype proportions between PCNSL (red) and sDLBCL (blue), with Wilcox test indicated (∗*p* < 0.05, ∗∗*p* < 0.01, and ∗∗∗*p* < 0.001). (D) The volcano plot shows the DEGs between diseases in M2 macrophages (including C0_M2_Mac_C1S, C2_M2_Mac_APOC1, C7_M2_Mac_NEAT1), red represents PCNSL, and blue represents sDLBCL. (E) The top five pathways of DEGs GO enrichment results in Figure D.

Comparative analysis revealed distinct distribution patterns of T cell subsets between sDLBCL and PCNSL. Specifically, the proportions of T0_Tnm_CCR7 (naive/central memory T cells) and T2_Treg_FOXP3 (regulatory T cells) were significantly higher in sDLBCL compared to PCNSL. Conversely, PCNSL exhibited increased frequencies of T1_Tcyto_NKG7 (cytotoxic T cells) and T3_Tpro_PKMYT1 (proliferating T cells) relative to sDLBCL (Figure 5A). These findings demonstrate fundamental differences in T cell composition between the two lymphoma types, suggesting distinct immune evasion mechanisms and antitumor immune responses in their respective microenvironments. Our investigation of cytotoxic T cells (Tcyto) in the tumor immune microenvironment, using TCellSI to quantify their functional states, revealed significant differences between PCNSL and sDLBCL. Notably, Tcyto cells in PCNSL exhibited the highest scores for terminal exhaustion among all evaluated populations, with levels significantly exceeding those observed in sDLBCL (Figure 5B).

Comprehensive analysis of myeloid cells identified eight distinct subclusters with specific phenotypic characteristics: C0, C2, and C7 represented M2-polarized macrophages (C0_M2_Mac_C1S, C2_M2_Mac_APOC1, C7_M2_Mac_NEAT1), C1 corresponded to monocytes, C3 to plasmacytoid dendritic cells (pDCs), C4 to lipid-associated macrophages (LAMs), C5 to conventional type 1 dendritic cells (cDC1), and C6 to cDC2 (Figure 5C). Quantitative comparison revealed significant enrichment of immunosuppressive M2 macrophage subsets (C0 and C7) in PCNSL compared to sDLBCL, while cDC1 cells (C5) were markedly more abundant in sDLBCL (Figure 5C).

Given the observed differences in M2 macrophage distribution, we performed differential gene expression analysis between PCNSL- and sDLBCL-derived M2 populations (Figure 5D). Pathway enrichment analysis of the top differentially expressed genes revealed fundamental functional distinctions: M2 macrophages in sDLBCL were prominently enriched for metabolic processes, including cellular respiration and oxidative phosphorylation, whereas their PCNSL counterparts showed significant involvement in MHC class II antigen presentation pathways (Figure 5E).

### Heterogeneity in immune cell interactions within the tumor microenvironment

**Figure 6:**
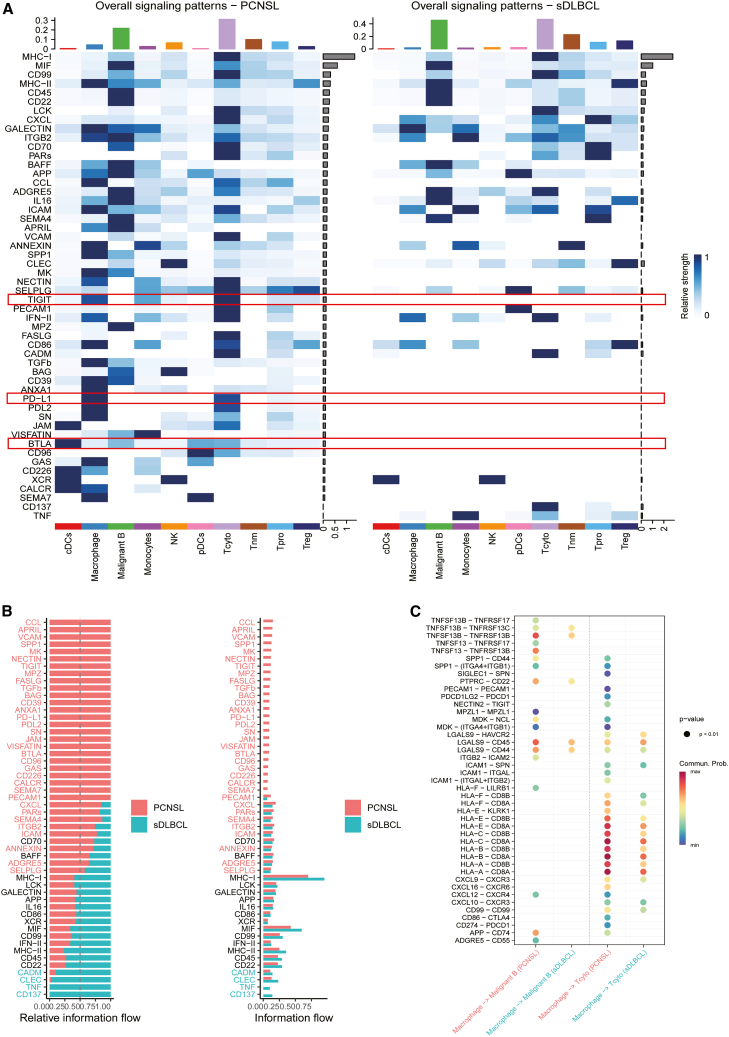
Differential cell-cell communication in PCNSL and sDLBCL (A) Heatmap of overall signaling patterns inferred by CellChat, with PCNSL shown on the left and sDLBCL on the right. Each row represents a signaling pathway, and each column a cell type; color intensity indicates relative communication strength. PCNSL exhibits a greater number of active (non-zero) signaling pathways than sDLBCL. (B) Comparison of signaling information flow between PCNSL (red) and sDLBCL (blue), shown as relative (left) and absolute (right) values. Overall signaling activity is higher in PCNSL. (C) Bubble plot illustrates macrophage interactions with malignant B cells and cytotoxic T cells in PCNSL and sDLBCL. Bubble size denotes statistical significance, and color represents communication probability.

Compared with sDLBCL, PCNSL displayed a greater number of detectable ligand-receptor signaling pathways mediating interactions between malignant B cells and immune cells (Figures 6A and 6B), indicating increased diversity of cell-cell communication. Notably, PCNSL exhibited a unique enrichment of key immune checkpoint interactions, namely, the co-inhibitory molecules PD-L1 and PDL2 (ligands for PD-1 that mediate T cell exhaustion), BTLA (a CD28 family inhibitor that dampens T cell activation), and TIGIT (a checkpoint that suppresses CD8^+^ T cell and NK cell function) (Figure 6A). Importantly, the overall cell interaction signals indicate that these checkpoint signals originate from different cellular compartments: PD-L1 and TIGIT interactions in PCNSL are primarily contributed by tumor microenvironment cells, including macrophages, monocytes, and cytotoxic T cells, while BTLA reflects bidirectional crosstalk between malignant B cells and immune cells (Figure 6A). These checkpoint interactions were accompanied by immunoregulatory molecules (CD226, CD9, CD39) and adhesion molecules (PECAM1, VCAM), while sDLBCL showed distinct TNF signaling and CD137 interactions (Figures 6B and 6C).

### Prognostic analysis of co-expression modules in PCNSL and sDLBCL

We analyzed three independent bulk transcriptome datasets: GSE34771 (PCNSL), GSE87371 (sDLBCL), and GSE10846 (sDLBCL). Using GSVA, we scored these datasets based on the top 25 hub genes from our 19 identified co-expression modules.

**Figure 7:**
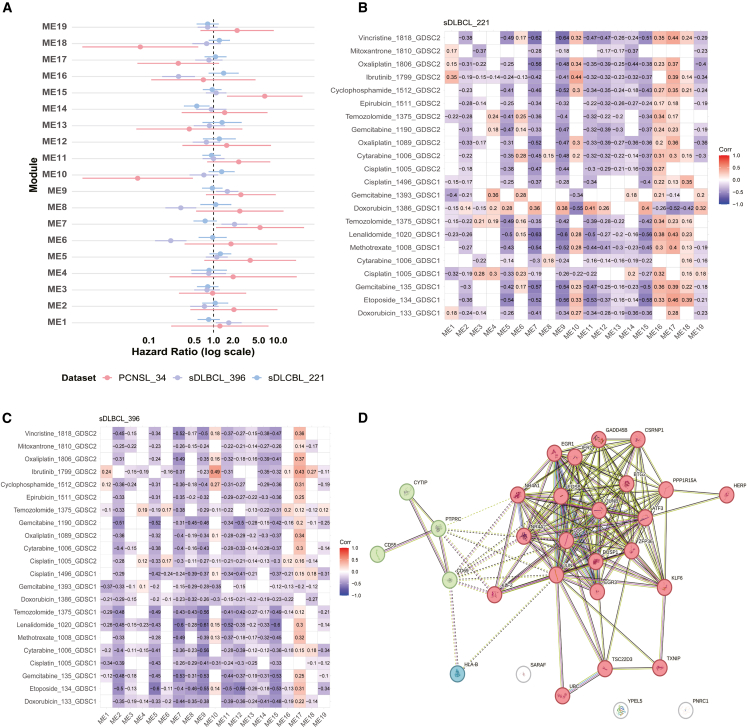
Discovery of prognostic and drug resistance modules (A) Results of prognostic analysis for 19 co-expression gene modules across three datasets: PCNSL (*N* = 34, red), sDLBCL-1 (*N* = 396, purple), sDLBCL-2 (*N* = 221, blue). The central point represents the Hazard Ratio (HR), with error bars indicating the 95% confidence interval. (B and C) The heatmap illustrates the correlation between the IC50 values of 22 drugs (representing drug sensitivity) and the scores of 19 gene co-expression modules in sDLBCL (B *N* = 221, C *N* = 396). Only statistically significant correlations (*p* < 0.05) are highlighted in color. (D) The network illustrates the protein-protein interactions (PPIs) among the 25 genes comprising ME17. PPIs were constructed using the STRING database.

Survival analysis revealed distinct prognostic patterns between PCNSL and sDLBCL. In PCNSL, high expression of ME7 and ME15 was associated with poor prognosis and increased risk, which were significantly upregulated in PCNSL compared to sDLBCL (Figure 4A). Conversely, low expression of ME10 and ME18 correlated with worse outcomes (Figure 7A). In sDLBCL, high expression of ME1, ME7, and ME9, along with low expression of ME6, ME8, ME14, and ME16, was linked to higher risk (Figure 7A).

### Drug resistance mechanisms by module-based pharmacogenomic analysis

Drug sensitivity analysis consistently revealed a significant positive correlation between ME17 module expression and drug IC50 values in two independent sDLBCL datasets (Figures 7B, 7C, and S4A–S4B). Notably, while ME17 showed the most consistent association across both cohorts, ME16 exhibited the strongest effect size in the sDLBCL-221 dataset. Protein-protein interaction analysis mapped a complex network in ME17, where FOS, JUN, and ATF3 emerged as top-ranking nodes based on topological centrality, pointing to a key regulatory node in chemoresistance (Figure 7D; Table S5). In contrast to sDLBCL, our analysis of a smaller PCNSL cohort (*N* = 34) did not reveal any expression modules (resistance modules) that were significantly positively correlated with drug sensitivity (Figures S4C–S4D), likely due to limited statistical power.

## Discussion

This study presents a systematic comparative single-cell analysis of PCNSL and sDLBCL, revealing fundamental differences in malignant programs, immune architecture, and transcriptional regulation across anatomical compartments. Our findings support a paradigm in which PCNSL represents a biologically distinct, tissue-adapted lymphoma subtype rather than a regional extension of systemic disease.

Compared with previous bulk transcriptomic studies, our single-cell approach captures the intratumoral complexity and cellular heterogeneity at an unprecedented resolution.^20^^,^^21^ Recent single-cell studies of systemic DLBCL have begun to resolve malignant diversification, but few have incorporated immune profiling or comparative CNS analyses.^22^^,^^23^ By integrating data from 31 patients and applying cross-sample harmonization, our framework provides a comprehensive view of malignant cell states and immune interactions across tissue contexts. This direct comparison uncovers divergence in transcriptional programs and microenvironments that bulk or disease-agnostic studies could not resolve.

We identified five malignant B-cell subtypes representing distinct functional states. Among them, the B0 subtype is preferentially enriched in PCNSL and exhibits a highly proliferative, progenitor-like transcriptional profile, consistent with previous reports.^24^ Its increased abundance in PCNSL may contribute to the elevated malignant cell fraction observed across B-cell populations in CNS tumors, supporting the notion that PCNSL develops under CNS-specific selective pressures.

Beyond malignant enrichment, B0 cells display intrinsic transcriptional features associated with mitotic progression and chromosome segregation, with the recurrent upregulation of cell-cycle and proliferation-related genes, including *TYMS*, *MKI67*, *MYBL2*, *RRM2*, and *ZWINT*, consistent with an actively proliferative state. As these findings are based on cross-sectional transcriptomic data, they support associative rather than causal interpretations. Whether the B0 program precedes CNS localization or reflects adaptation to the CNS microenvironment remains unresolved and will require longitudinal and functional studies.

Concurrently, the immune microenvironment of PCNSL is characterized by a high abundance of exhausted cytotoxic T cells, immunosuppressive myeloid populations, and limited memory T cell responses, in stark contrast to the immune-active landscape observed in systemic DLBCL. This “proliferative-suppressive” dual axis defines a conceptual model of PCNSL pathogenesis, consistent with the CNS’s unique structural and immunological constraints.^17^^,^^25^

Intercellular communication analysis revealed dominant immunosuppressive features in PCNSL, involving synergistic engagement of PD-L1, PD-L2, BTLA, and TIGIT. Recent evidence highlights that, beyond total expression levels, the differential expression of individual transcript variants of PD-1 and PD-L2 (linked to Th1/Th2 status) is critical for prognosis prediction in PCNSL, adding a layer of complexity to these checkpoint interactions.^26^ These may drive both T cell and NK-cell dysfunction.^27^^,^^28^ In contrast, sDLBCL is dominated by TNF and CD137 signaling, displaying a more pronounced pro-inflammatory and co-stimulatory profile that may confer greater sensitivity to immunotherapy.^29^^,^^30^ The intricate immunoregulatory network in PCNSL, including CD39-mediated adenosine generation, along with adhesion molecules such as PECAM1 and VCAM, further facilitates immune evasion.^31^^,^^32^ These findings suggest that PCNSL may require combinatorial immune checkpoint blockade, whereas sDLBCL might be more amenable to strategies such as CD137 agonism. Future functional validation is warranted to guide personalized therapeutic approaches.

Distinct co-expression networks between PCNSL and sDLBCL highlight fundamental differences in their molecular pathogenesis, with PCNSL displaying enhanced proliferative and angiogenic signatures compared to the immune-interaction signature of sDLBCL. Furthermore, hierarchical module expression across B-cell subtypes provides insights into their potential developmental relationships and functional diversity in these malignancies. Mechanistically, our results support a model in which CNS-resident malignant B cells undergo selective transcriptional reprogramming to sustain proliferation and evade immune surveillance. Co-expression modules enriched in PCNSL are characterized by cell cycle and MYC activation, features that are strongly associated with poor prognosis.^33^ Enrichment in MYC-regulated gene modules and VEGF signaling also suggests that the B0 subtype may drive angiogenesis and tissue invasion within the immune-privileged CNS microenvironment.^34^^,^^35^

Methodologically, we employed a multi-layered pipeline combining single-cell transcriptomics, co-expression network modeling, survival association, and drug sensitivity prediction. In PCNSL, ME7 and ME15 modules were prognostically significant and linked to B0-like states. In sDLBCL, ME17 was associated with drug resistance, characterized by a dense PPI network. Although FOS emerged as a central node, our topological analysis further highlights the critical contributions of other high-degree hubs, such as JUN, ATF3, and EGR1.^36^^,^^37^ FOS strongly interacts with JUN family members, highlighting the central role of the Activator Protein-1 complex.^38^ Rather than acting alone, FOS likely cooperates to drive a rapid transcriptional response to chemotherapy, suggesting that sDLBCL drug resistance relies on a coordinated immediate-early gene program rather than a single effector. Given the limited sample size in the PCNSL bulk cohort, these associations should be interpreted with caution, and the prognostic and drug-resistance findings are presented as exploratory and hypothesis-generating rather than definitive. These results demonstrate the clinical relevance of transcriptionally defined cell states in predicting therapeutic response, particularly in PCNSL where conventional chemotherapy remains inadequate.^39^^,^^40^

Our findings hold broader implications for lymphoma biology and oncology. They support a paradigm shift in conceptualizing CNS lymphomas, from an anatomically based subclassification toward a molecularly defined, tissue-adaptive disease model.^41^ This aligns with an emerging perspective in cancer biology that advocates for classifying tumors based on evolutionary trajectories and microenvironmental adaptation rather than solely on organ of origin.^42^^,^^43^ Our results thus offer a framework applicable beyond PCNSL, extending to other CNS malignancies. Distinct from the previous landscape study, our work leverages a harmonized cross-disease atlas of PCNSL and sDLBCL to drive novel discoveries.^44^^,^^45^ Beyond a descriptive inventory, this joint analysis highlights the prognostic B0 subtype and contrasts the strikingly divergent immune niches, revealing specific immunosuppressive states unique to the CNS. Coupled with our identification of resistance-driving modules, this study shifts the focus from atlas characterization to defining actionable therapeutic targets.

### Limitations of the study

Despite these advances, limitations remain. The rarity of PCNSL restricted patient sample size and resulted in an unavoidable imbalance between the PCNSL and sDLBCL cohorts, which may affect the patient-level generalizability of certain comparisons. Publicly available bulk datasets were limited for PCNSL, and sDLBCL cohorts showed variable reproducibility. This study is primarily transcriptomic and observational in nature. Although cross-cohort and cross-platform validation was performed, direct functional perturbation experiments were not feasible due to limited biopsy material. Therefore, causal relationships between identified cell states and therapeutic resistance require further experimental validation. Future work should include multicenter, longitudinal cohorts and incorporate spatial transcriptomics to better resolve anatomical context. Functional validation in model systems, including CRISPR perturbation and lineage tracing, will be essential to confirm causal mechanisms. Further integration of epigenomic and proteomic data may clarify regulatory dynamics, while pre/post-treatment sampling could reveal how tumor and immune compartments remodel under therapy. Comparative analyses with gliomas and CNS metastases may identify shared adaptation strategies and therapeutic targets.

In conclusion, this study redefines PCNSL as a transcriptionally, immunologically, and clinically distinct subtype of DLBCL. By delineating progenitor-like malignant states, immune evasion networks, and subtype-specific resistance mechanisms, we provide a roadmap for developing molecularly guided, tissue-adapted therapeutic strategies in CNS lymphomas.

## Resource availability

### Lead contact

Requests for further information and resources should be directed to and will be fulfilled by the lead contact, Junfang Chen (junfang_chen@fudan.edu.cn).

### Materials availability

Due to ethical and institutional policy restrictions, access to human tumor samples and related materials generated in this study may be limited. Material requests should be made directly to the principal investigator and reviewed by the Ethics Committee of the Affiliated Hospital of Hebei University.

### Data and code availability

• The single-cell RNA sequencing dataset generated from our in-house cohort (PCNSL, n = 5) will be shared by the lead contact (junfang_chen@fudan.edu.cn) upon request.
• Publicly available datasets used in this study have been previously published, and their accession codes (GEO: GSE87371, GSE10846, GSE34771; CNGBdb: CNP0001940; Zenodo: 7813151) are listed in the key resources table.
• Original code reported in this paper is available from the lead contact upon request.
• Any additional information required to reanalyze the data reported in this paper is available from the lead contact upon request.

## Acknowledgments

The authors gratefully thank the patients and volunteers for their contributions to sample collection. Graphical abstract was created using Figdraw. This work was supported by a startup grant from the Greater Bay Area Institute of Precision Medicine (Guangzhou) to J.C. (grant no. I0007), the 10.13039/501100001809National Natural Science Foundation of China (grant no. 32370639), and the 10.13039/501100003453Natural Science Foundation of Guangdong Province (grant no. 2024A1515012116). Y.J. was supported by the Cultivation Project of Precision Medicine Joint Fund of Hebei Natural Science Foundation (H2025201063).

## Author contributions

F.C.: conceptualization, methodology, software, formal analysis, investigation, data curation, writing – original draft, and visualization. X.W.: conceptualization, validation, formal analysis, investigation, data curation, writing – original draft, and visualization. S.Z.: methodology, software, and formal analysis. Y.L.: validation, investigation, and data curation. S.W.: software, validation, and data curation. J.Z.: resources and investigation. P.L.: resources and investigation. Z.H.: validation and visualization. W.L.: resources and investigation. Z.S.: resources and data curation. C.T.: validation and investigation. Y.L.: resources and investigation. G.H.: resources and validation. J.C.: conceptualization, resources, writing – review and editing, supervision, project administration, and funding acquisition. F.L.: conceptualization, resources, writing – review and editing, supervision, project administration, and funding acquisition. Y.J.: conceptualization, resources, writing – review and editing, supervision, project administration, and funding acquisition.

## Declaration of interests

The authors declare no competing interests.

## Declaration of generative AI and AI-assisted technologies in the writing process

During the preparation of this work, the authors used ChatGPT in order to improve readability and language. After using this tool/service, the authors reviewed and edited the content as needed and take full responsibility for the content of the published article.

## STAR★Methods

### Key resources table

**Table:** 

REAGENT or RESOURCE	SOURCE	IDENTIFIER
**Biological samples**		

Primary central nervous system lymphoma (PCNSL) tumor tissues (*N* = 5)	Affiliated Hospital of Hebei University	N/A

**Chemicals, peptides, and recombinant proteins**		

MACS Tissue Storage Solution	Miltenyi Biotec	Cat# 130-100-008
10x Genomics Chromium™ platform reagents	10x Genomics	N/A

**Deposited data**		

Single-cell RNA sequencing data of in-house PCNSL cohort (*N* = 5)	This paper	N/A
Single-cell RNA sequencing data of sDLBCL (*N* = 17)	Ye et al.^45^	CNGBdb: CNP0001940
Single-cell RNA sequencing data of PCNSL (*N* = 9)	Liu et al.^44^	Zenodo: 7813151
Bulk transcriptomic data of PCNSL (*N* = 34)	Kawaguchi et al.^46^	GEO: GSE34771
Bulk transcriptomic data of sDLBCL (*N* = 396)	Lenz et al.^47^	GEO: GSE10846
Bulk transcriptomic data of sDLBCL (*N* = 221)	Dubois et al.^48^	GEO: GSE87371

**Software and algorithms**		

R (v4.3.3)	R Foundation	https://www.r-project.org/
Seurat (v5.1.0)	Hao et al.^49^	https://satijalab.org/seurat/
DoubletFinder (v2.0.4)	McGinnis et al.^50^	https://github.com/chris-mcginnis-ucsf/DoubletFinde
Harmony (v0.1.1)	Korsunsky et al.^51^	https://github.com/immunogenomics/harmony
Monocle 2 (v2.30.1)	Trapnell et al.^52^	http://cole-trapnell-lab.github.io/monocle-release/
CytoTRACE2 (v1.0.0)	Gulati et al.^53^	https://cytotrace.stanford.edu
hdWGCNA (v0.3.3)	Morabito et al.^54^	https://smorabit.github.io/hdWGCNA/
CellChat (v1.5.0)	Jin et al.^55^	https://github.com/sqjin/CellChat
TCellSI (v1.2.0)	Yang et al.^56^	https://github.com/ZiyiLiang/TCellSI
CIBERSORTx	Newman et al.^57^	https://cibersortx.stanford.edu/
GSVA (v1.50.5)	Hänzelmann et al.^58^	https://bioconductor.org/packages/GSVA/
oncoPredict (v1.2)	Maeser et al.^59^	https://github.com/oscar-franks/oncoPredict
STRING database (v12.0)	Szklarczyk et al.^60^	https://string-db.org
Metascape	Zhou et al.^61^	https://metascape.org
clusterProfiler (v4.10.1)	Wu et al.^62^	https://bioconductor.org/packages/clusterProfiler/
inferCNV (v1.18.1)	Tirosh et al.^63^	https://github.com/broadinstitute/inferCNV

**Other**		

Genomics of Drug Sensitivity in Cancer (GDSC) database	Yang et al.^64^	https://www.cancerrxgene.org/

### Experimental model and study participant details

#### Ethics approval and consent for the use of human specimens

Human ethics was approved by Ethics Committee of the Affiliated Hospital of Hebei University (HDFYLL-KY-2023-087).

#### Study participants

This study cohort included five patients with primary central nervous system lymphoma (Table S1), who were recruited from the Affiliated Hospital of Hebei University between July 2023 and December 2024.

All participants or their legal guardians provided written informed consent prior to enrollment.

### Method details

#### Study cohort

This study utilized scRNA-seq data from three independent cohorts: (1) an in-house cohort consisting of treatment-naïve tumor tissues from 5 PCNSL cases; (2) an approved-access cohort of 17 sDLBCL cases^45^; and (3) a publicly available dataset comprising 9 PCNSL samples.^44^ Detailed clinical characteristics are provided in Tables S1 and S2.

#### Sample collection

A single biopsy core per lesion was obtained via stereotactic needle biopsy (16G coaxial system, Medtronic StealthStation S8 guidance). Immediately after intraoperative frozen section confirmation, ≥30 mg viable tissue was preserved in MACS Tissue Storage Solution (Miltenyi Biotec, 130-100-008) at 4°C for single-cell sequencing. All specimens met stringent quality thresholds (≥95% viability) and were anonymized with unique identifiers (PCNSL-001 to PCNSL-005). Written informed consent was obtained in compliance with the Declaration of Helsinki.

#### Single-cell library preparation

Single-cell capture and barcoding were performed using the 10x Genomics Chromium platform, where cells, barcoded gel beads (containing 16-nt 10x Barcode, 10-nt UMI, and 13-nt Switch Oligo), and reverse transcription reagents were co-encapsulated into Gel Bead-In-Emulsions (GEMs) via a microfluidic chip. Limiting dilution ensured single-cell resolution (1 cell per 10 GEMs), with subsequent GEM dissolution and cell lysis releasing oligonucleotides for first-strand cDNA synthesis (45°C, 120 min) using poly(dT) primers. Post-reaction mixtures were pooled and purified with silane magnetic beads (0.6× ratio) to remove residual reagents, followed by full-length cDNA amplification via 12-cycle PCR with universal primers targeting constant regions.

#### Quality control of scRNA-seq data

We performed comprehensive integration of all single-cell RNA sequencing datasets, encompassing 14 PCNSL and 17 sDLBCL cases, using the Seurat R package (v5.1.0).^49^ Data processing included rigorous quality control measures: cells were retained only if they met the following criteria: Gene counts (nFeature_RNA) between 500 and 6000, Mitochondrial gene content (percent.mt) < 15%, Total UMI counts (nCount_RNA) < 50 000. To address potential technical artifacts, we employed DoubletFinder (v2.0.4) ^50^ for systematic identification and removal of doublets. Following these stringent quality control procedures, our final high-quality dataset comprised 171 322 cells and 17 476 genes, providing a robust foundation for subsequent analyses, and the detailed information is shown in Tables S1 and S2.

#### Dimensionality reduction and clustering

Following quality control, gene expression counts were normalized to 10 000 transcripts per cell via total count normalization with log-transformation. The top 3 000 highly variable genes (HVGs) were selected using variance-stabilizing transformation. Principal component analysis was applied to scaled expression data, and the first 30 principal components (PCs) were retained for downstream analysis. Technical batch effects were mitigated using Harmony (v0.1.1), ^51^ preserving biological variation. The harmonized PCs were used to compute UMAP embeddings and perform unsupervised clustering, enabling cell population identification free of technical bias.

#### Cell type annotation

Following dimensionality reduction, we performed Louvain clustering (resolution = 0.2) to identify major cell populations, including NK cells (*KLRD1*+), T cells (*CD3D*+), B cells *(MS4A1*+), myeloid cells (*LYZ*+), oligodendrocytes (*MBP*+), cancer-associated fibroblasts (CAFs; *COL1A1*+), and endothelial cells (*MCAM*+) (Figure S1A). Cluster-specific marker genes were identified using Seurat’s FindAllMarkers function (logFC threshold >0.25, adjusted *p* < 0.05). Given their synergistic roles in tumor microenvironment (TME) organization, we aggregated oligodendrocytes (CNS-specific), CAFs, and endothelial cells into a unified stromal compartment to enable cross-disease comparison of PCNSL and sDLBCL microenvironments. Subsequently, we performed subclustering on lymphoid (B/T cells) and myeloid populations to delineate functionally distinct subsets, see Figure S1 for details.

#### Malignant B-cell identification via cross-disease CNV profiling

To delineate malignant B-cell populations, we performed disease-specific inference of copy number variations (CNVs) using inferCNV (v1.18.1).^63^ Specifically, we utilized the i6 hidden Markov model (HMM), estimating six copy-number states ranging from complete loss (State 1) to high-level amplification (State 6), calibrated against reference immune cells. An expression cutoff of 0.1 was applied. Malignant B-cells were rigorously defined as those exhibiting characteristic genomic instability signatures: high proportions of State 1 (deep deletion) or States 5–6 (amplifications, modeled as ≥2 copies). All other B-cells dominated by copy-number neutral regions (State 3) were classified as non-malignant. Malignancy propensity of each subtype was quantified as the ratio of malignant-to-normal B-cells.

#### Pathway enrichment analysis

Pathway enrichment analysis was performed using the clusterProfiler R package (v4.10.1) to identify significantly enriched biological pathways. ^62^ The top 50 differentially expressed genes (DEGs) of B cell subtypes were used as input and enriched against the Gene Ontology (GO) database.^65^ The Metascape online tool (https://metascape.org) was used to perform pathway enrichment on all hub genes in the PCNSL and sDLBCL high-expression gene modules, integrating multiple databases (KEGG, Reactome, GO) for comprehensive functional annotation.^61^

#### Developmental trajectory analysis of B-cell subtypes

To assess differentiation potential and infer developmental trajectories among malignant B-cell subtypes, we applied CytoTRACE2 (v1.0.0).^53^ This computational approach quantifies cellular differentiation states, enabling comparative analysis of differentiation capacity across distinct B-cell subpopulations. For orthogonal trajectory inference, Monocle 2 (v2.30.1) was applied.^52^ To optimize computational efficiency, malignant B cells were randomly downsampled to 1,000 cells per subtype (B0-B4). Dimensionality reduction was performed using the DDRTree algorithm based on highly dispersed genes. Pseudotime was inferred with the progenitor-like B0 subtype explicitly specified as the root state.

#### T cytotoxic cell exhaustion profiling

To systematically evaluate T cell dysfunction states, we applied TCellSI (v1.2.0) to quantify cytotoxicity, progenitor exhaustion, terminal exhaustion, and senescence scores in T cytotoxic cells.^56^ These metrics were comparatively analyzed between PCNSL and sDLBCL microenvironments to identify disease-specific exhaustion patterns.

#### Cell chat analysis between PCNSL and sDLBCL

We used CellChat (v1.5.0)^55^ to compare cell-cell communication networks between TMEs. After quality control and normalization, two TME datasets were processed using the default workflow with the human CellChatDB for ligand-receptor interactions. Communication probabilities were computed at the molecular (computeCommunProb) and pathway (computeCommunProbPathway) levels to identify differential signaling between malignant B cells and non-malignant immune cells. Signal pattern represents the overall communication probability of each ligand-receptor pathway, summed across all cell types, while relative information flow quantifies each pathway’s contribution to the total communication network, computed as normalized probabilities; absolute signaling strength was also included to indicate signal intensity.

#### Gene co-expression network analysis

We performed weighted gene co-expression network analysis on rigorously defined malignant B-cells (*N* = 70,934) using hdWGCNA (v0.3.3). ^54^ Following normalization and HVG selection, an optimal soft-thresholding power of 12 was selected based on the scale-free topology criterion (Figure S4A). A signed adjacency matrix was transformed into a topological overlap matrix (TOM), and modules were identified via dynamic tree cutting. Genes were ranked within each module by module eigengene-based connectivity (kME) to identify highly connected hub genes, extracting the top 25 for downstream analyses.

#### Deconvolution of bulk transcriptomes

To infer cellular composition in bulk tumor tissues, we employed CIBERSORTx.^57^ A custom single-cell reference signature matrix was constructed by randomly downsampling 500 cells per annotated subcelltype from our single-cell dataset to prevent dominant cluster bias. This matrix was utilized to deconvolute bulk RNA-seq data from the PCNSL cohort (*N* = 34) and two sDLBCL cohorts (*N* = 221 and *N* = 396). The relative fractions of each cell subset were estimated using 100 permutations for significance assessment.

#### Gene set variation analysis (GSVA) of co-expression modules

Gene expression profiles were obtained from three independent GEO datasets: GSE34771 (PCNSL, *N* = 34),^46^ GSE87371 (sDLBCL, *N* = 221),^66^ and GSE10846 (sDLBCL, *N* = 396).^47^ We quantified module activity patterns in bulk transcriptomes using GSVA (v1.50.5).^58^ Custom gene sets were defined as the top 25 hub genes from each co-expression module identified through hdWGCNA. Enrichment scores were calculated using a Gaussian kernel (kcdf = “Gaussian”) to estimate relative pathway activity across samples.

#### Survival analysis

Clinical data including overall survival (OS) were extracted from corresponding GEO metadata. Using the survival R package (v3.5.8),^67^ we performed univariate Cox proportional hazards regression based on gene co-expression module scores to identify co-expression modules significantly associated with OS (*p* < 0.05), with age and sex included as covariates in the model. All analyses were conducted in R (v4.3.3).

#### Drug sensitivity analysis

Drug sensitivity was computationally predicted using the oncoPredict R package (v1.2), ^59^ which infers therapeutic responses from transcriptomic profiles by leveraging the Genomics of Drug Sensitivity in Cancer (GDSC) database as a ref. ^64^ The algorithm estimates the half-maximal inhibitory concentration (IC50) for each drug available in GDSC, quantifying the sensitivity of individual samples based on their gene expression patterns. Default parameters were applied for all predictions. Drugs were selected as the intersection between compounds with available response data in the GDSC database and agents with established or potential clinical use in PCNSL and/or systemic DLBCL (Table S4). To evaluate associations between drug sensitivity and module activity, partial correlation analyses were performed between IC50 values and module scores, while adjusting for age and sex as covariates. Statistical significance was defined as *p* < 0.05.

#### Protein-protein interaction (PPI) network construction

We utilized the STRING database (v12.0; https://string-db.org)^60^ to construct a protein-protein interaction network for hub genes within drug-resistant gene modules, aiming to identify core proteins in the modules and infer potential drug resistance mechanisms.

### Quantification and statistical analysis

Statistical differences between independent groups were evaluated using the two-sided Wilcoxon rank-sum test. Where applicable, *p*-values were adjusted for multiple comparisons to control the false discovery rate (FDR). Asterisks denote statistical significance as follows: ∗*p* < 0.05, ∗∗*p* < 0.01, ∗∗∗*p* < 0.001, ∗∗∗∗*p* < 0.0001, and ns indicates not significant. Comprehensive statistical details for all relevant figures, including exact *p*-values, adjusted *p*-values, test statistics, and exact sample sizes (N), are provided in Table S10.

## Supplemental information

Document S1. Figures S1–S8 and Tables S1–S5

Table S6. Summary of the counts of cell subtypes in the sample

Table S7. Summary of B cell subtype top 50 DEGs

Table S8. Summary of 25 hub genes in gene co-expression modules

Table S9. Estimated cellular fractions of B-cell subsets in PCNSL and sDLBCL cohorts derived from CIBERSORTx deconvolution

Table S10. Detailed statistical information for Figures 2–5
