# Supplementary material for: Single cell profiling reveals malignant states and immune landscapes in PCNSL and systemic DLBCL
Source: iScience. 2026 Apr 2;29(5):115563. doi: 10.1016/j.isci.2026.115563 (PMC13097095; doi:10.1016/j.isci.2026.115563)
Supplement: Document S1. Figures S1–S8 and Tables S1–S5 [file mmc1.pdf]

iScience, Volume *29*

Supplemental information

Single cell profiling reveals malignant states and immune landscapes in PCNSL and systemic DLBCL

Fuqiang Cai, Xiaofang Wang, Shunjie Zhang, Yumiao Li, Shenghan Wang, Jijun Zhu, Pan Li, Zhongting Huang, Weixin Liu, Zhijian Song, Chang Tian, Yan Li, Guiliang Han, Junfang Chen, Fei Ling, and Youchao Jia

Supplementary Materials

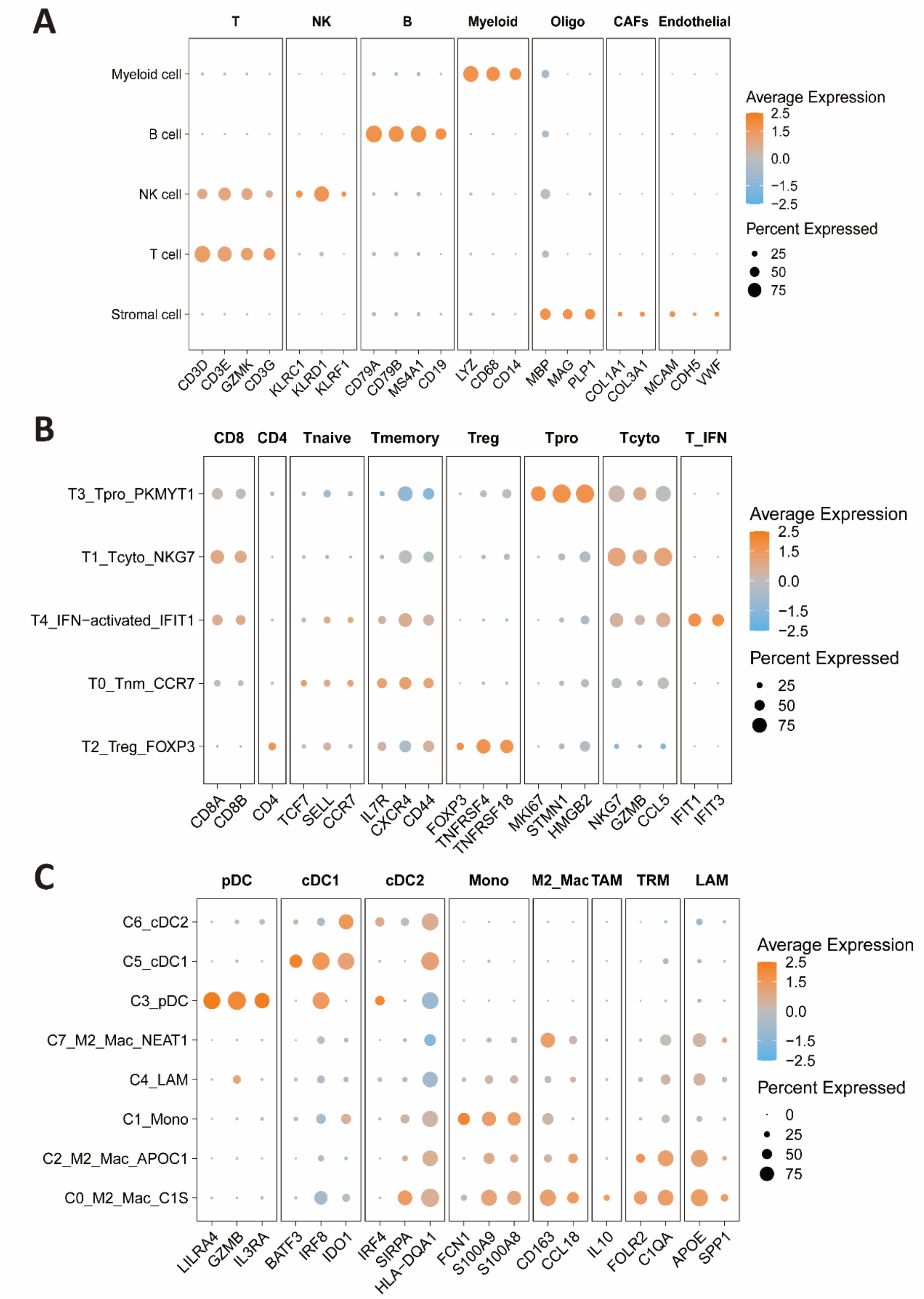

Supplemental Figure S1. Annotation of cell clusters using lineage-specific marker genes. A-C

Scaled average expressions (Average Expression) and proportion of cells expressing (Percent

Expressed) the canonical markers used to define the clusters(all cells, T cells, myeloid cells). For the clustering analysis of B cells, the top 30 principal components (PCs) were selected based on 3000 highly variable genes (HVGs) with a resolution of 0.1, and clusters containing fewer than 150 cells were excluded, leaving subpopulations B0 to B4. For T cells, the same strategy of selecting top 30 PCs from 3000 HVGs was applied but with a resolution of 0.2, identifying five distinct subpopulations: T0_Tnm (central memory T cells) marked by *CCR7*, *IL7R*, and *CXCR4*; T1_Tcyto (cytotoxic T cells) characterized by *NKG7*, *GZMB*, and *CCL5*; T2_Treg (regulatory T cells) defined by *FOXP3*, *TNFRSF4*, and *TNFRSF18*; T3_Tpro (proliferative T cells) marked by *MKI67*, *STMN1*, and *HMGB2*; and T4_IFN_activated (IFN-activated T cells) identified by *IFIT1* and *IFIT3* (Supplementary Fig. S1B). For myeloid cells, clustering with top 30 PCs from 3000 HVGs at a resolution of 0.1 yielded eight subpopulations: M2-macrophages expressing *CD163* and *CCL18*; monocytes defined by *FCN1*; pDC (plasmacytoid dendritic cells) characterized by *LILRA4*, *GZMB*, and *IL3RA*; LAM (lipid-associated macrophages) marked by *APOE* and *SPP1*; cDC1 (conventional dendritic cells 1) defined by *BATF3*, *IRF8*, and *IDO1*; and cDC2 (conventional dendritic cells 2) expressing *IRF4* and *HLA-DQA1* (Supplementary Fig. S1C).

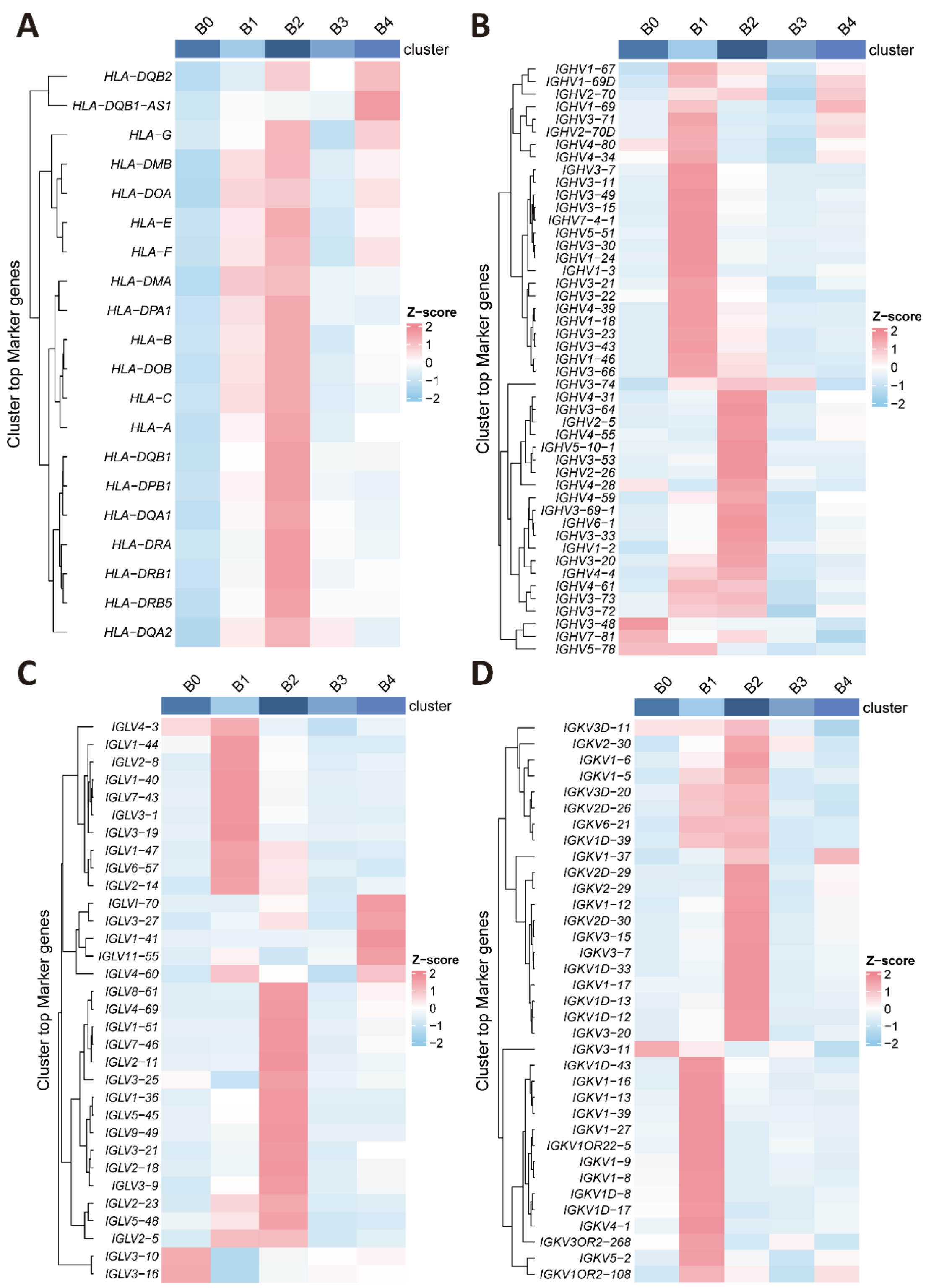

Supplemental Figure S2. HLA and immunoglobulin gene expression across B-cell subtypes.

A-D Heatmap of average expression levels of HLA (human leukocyte antigen) and immunoglobulin (IG) gene families in different B cell subtypes. The gene expression levels were transformed by z-score.

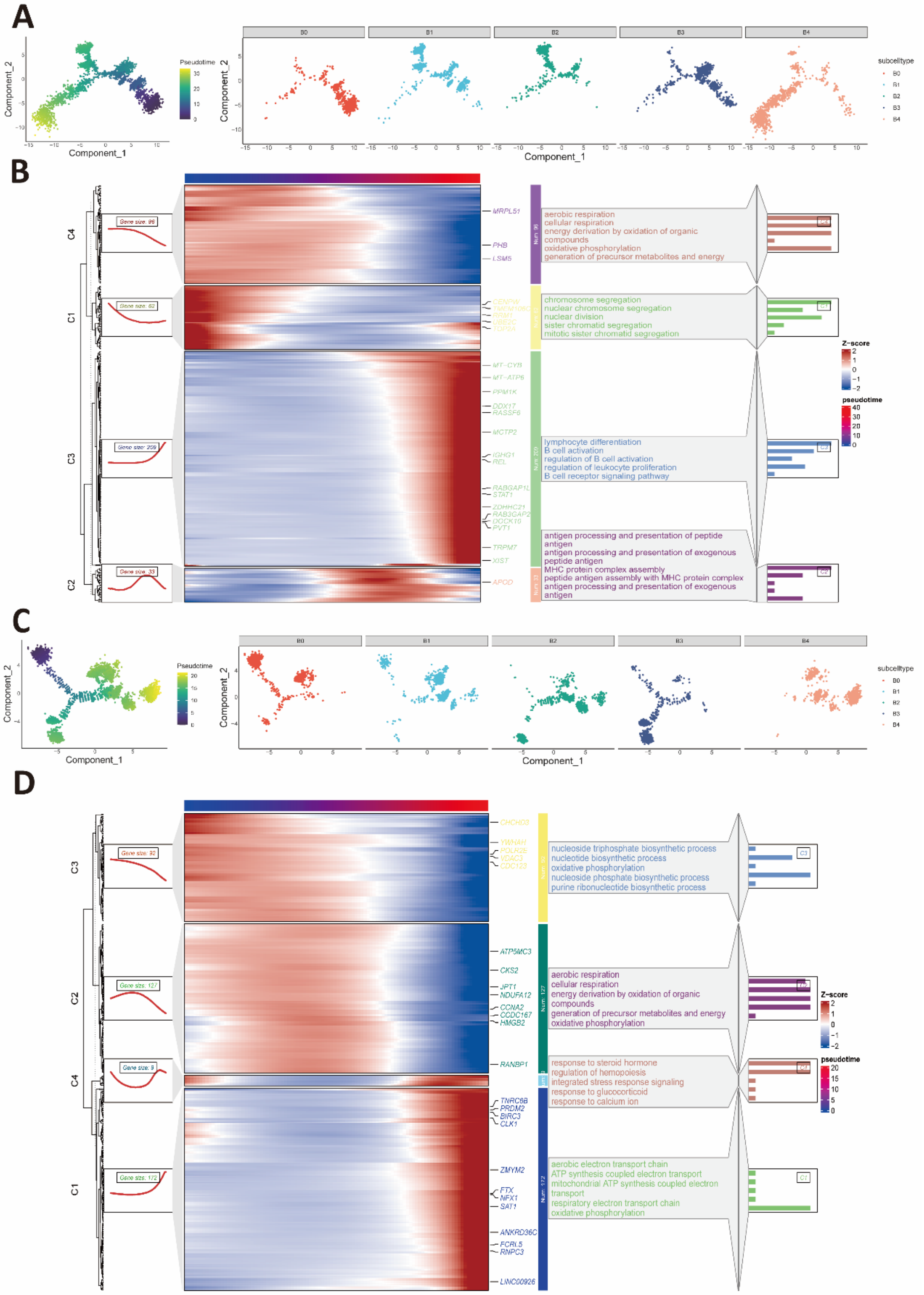

Supplemental Figure S3. Trajectory inference of malignant B cells in PCNSL and sDLBCL. A-B trajectory analysis of malignant B-cell subtypes in PCNSL. Panel A shows the pseudotime

trajectory and distribution of B0-B4 subtypes, while panel B displays pseudotime-ordered gene expression dynamics and corresponding functional modules, highlighting sequential programs related to proliferation, chromosome segregation, immune regulation, and antigen presentation. **C-D** corresponding trajectory and pseudotime-resolved transcriptional programs in sDLBCL. Compared with PCNSL, sDLBCL exhibits distinct pseudotime-associated gene modules predominantly enriched for metabolic and bioenergetic processes.

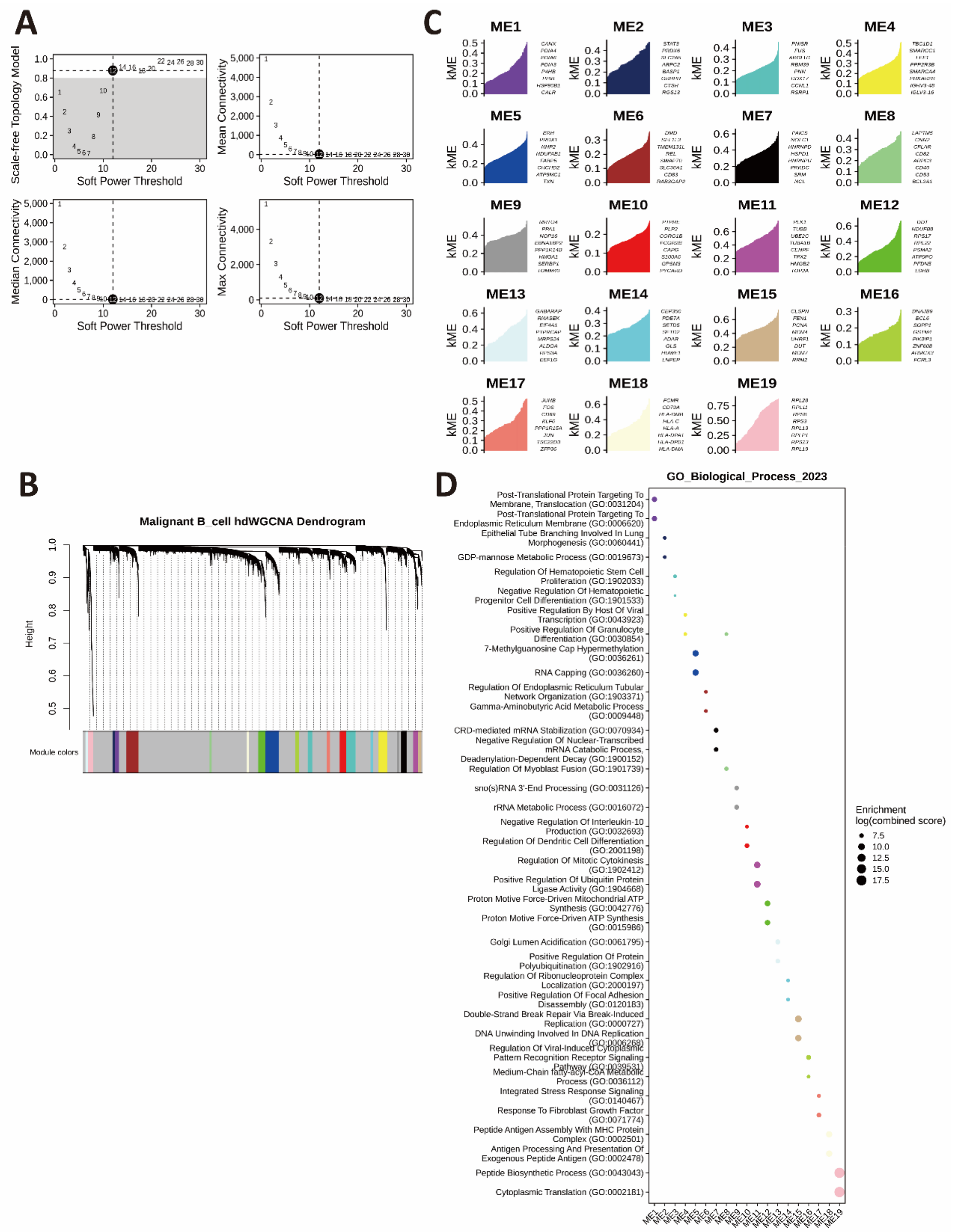

Supplemental Figure S4. Construction of the hdWGCNA co-expression network in malignant B cells. A Scale-free topology model fit index (signed R²) and network connectivity metrics (mean, median, and maximum connectivity) were evaluated across a range of soft-thresholding powers. A power of 12 was selected, at which the network approximated a scale-free topology while maintaining reduced connectivity. **B** Hierarchical clustering dendrogram of

genes expressed in malignant B cells based on topological overlap. Gene modules were identified using dynamic tree cutting and are shown by the color bar beneath the dendrogram. Genes assigned to the gray module were not grouped into any specific module and were excluded from downstream analyses. **C** Module eigengene-based connectivity (kME) was calculated for each gene across all identified hdWGCNA modules. For each module (ME1-ME19), genes are ranked by decreasing kME values, reflecting their correlation with the corresponding module eigengene. Representative high-kME genes are shown for each module, highlighting putative hub genes that may play central roles in module-specific transcriptional programs. **D** The bubble chart shows the top 2 pathways of the enrichment results of hub25 genes in 19 gene co-expression modules based on GO_Biological_Process_2023. The bubble colors represent different modules, and the bubble sizes represent the enrichment log (combined score).

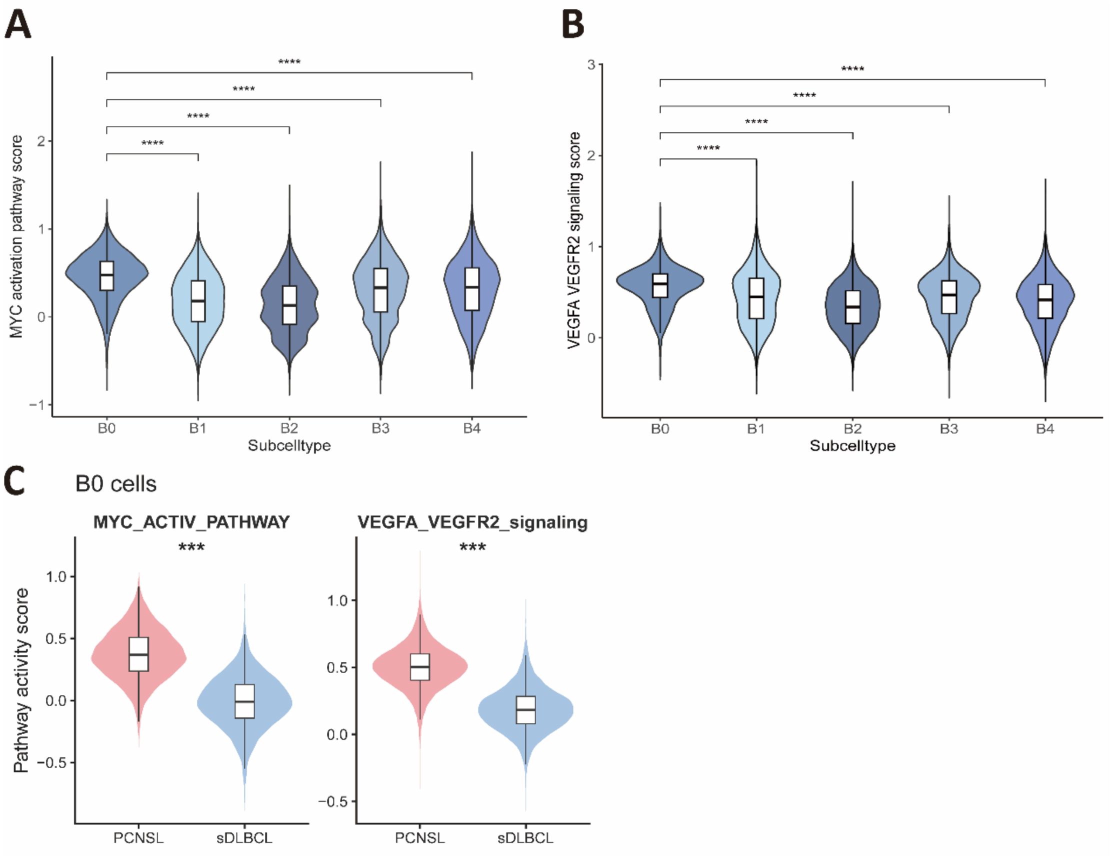

Supplemental Figure S5. Pathway activity scoring reveals distinct oncogenic signaling in the progenitor-like B0 subtype. A-B Violin plots displaying the single-cell pathway activation scores for **A** MYC activation pathway and **B** VEGFA-VEGFR2 signaling across the five identified malignant B-cell subtypes (B0-B4). The B0 subtype exhibits significantly elevated activity in both pathways compared to other clusters (**** $P < 0.0001$). **C** Comparative analysis of pathway scores specifically within the B0 subtype derived from PCNSL (red) versus sDLBCL (blue). B0 cells from PCNSL show significantly higher activation of MYC and VEGFA signaling compared to their sDLBCL counterparts (*** $P < 0.001$). Statistical significance was determined using the Wilcoxon rank-sum test.

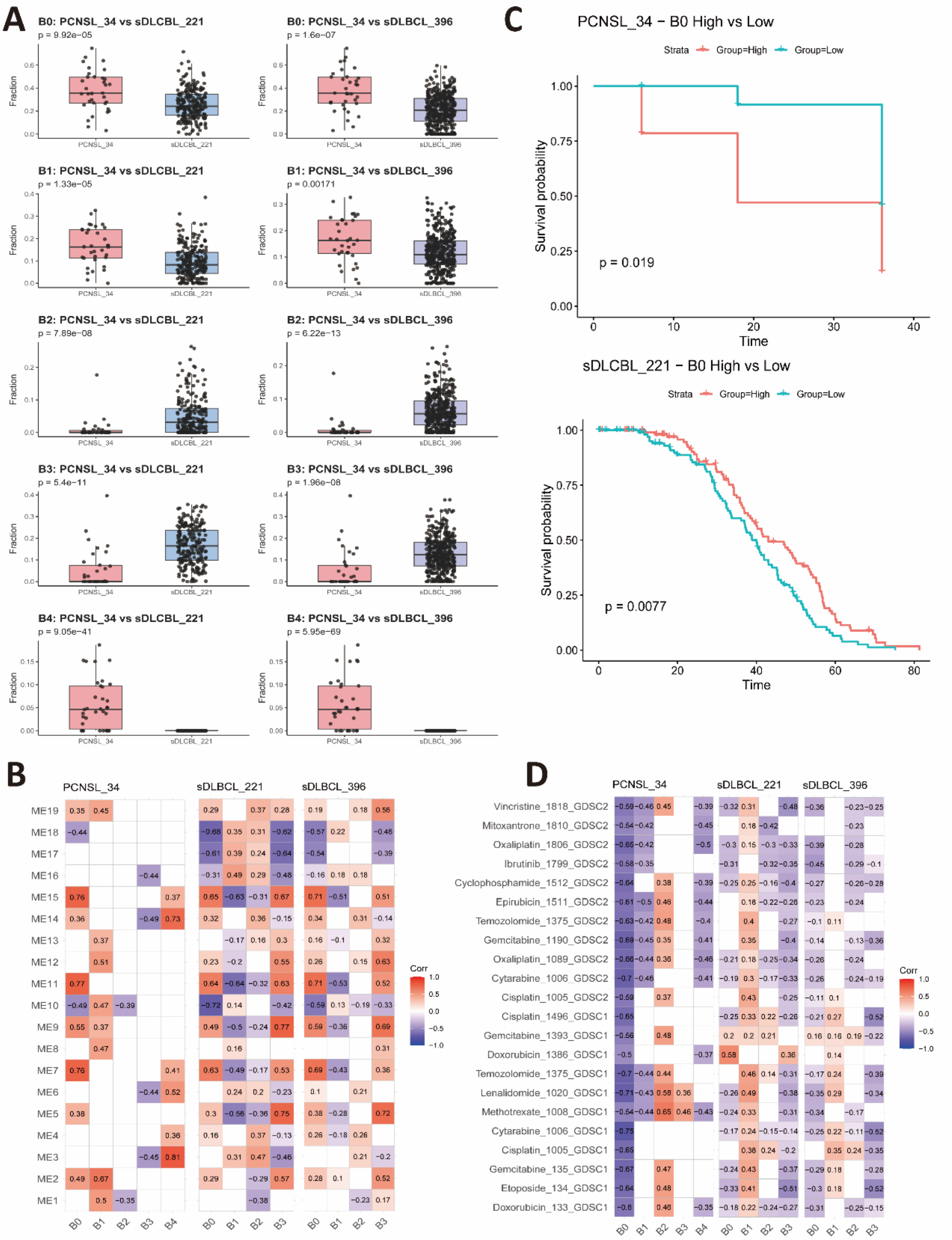

Supplemental Figure S6. Distinct cellular landscape, functional correlation, and clinical significance of B-cell subsets in PCNSL and sDLBCL. A Comparison of estimated cell fractions (B0-B4) between PCNSL (N=34) and sDLBCL cohorts (N=221, N=396) using CIBERSORTx deconvolution based on single-cell signatures. Box plots show the distribution of cell proportions; P-values indicate significance (Wilcoxon test). **B** The heatmap shows the partial

correlation analysis between the proportion of B cell subsets and co-expression module scores (ME) in the three cohorts. Only significant correlations (P < 0.05) are colored in the figure. **C** Kaplan-Meier survival curves stratified by B0 fraction (High vs. Low) in PCNSL and sDLBCL cohorts. Log-rank tests were used to determine significance. **D** Partial correlation analysis linking B-cell subset fractions to predicted drug sensitivity (IC50) for various chemotherapeutic agents. Blank cells indicate non-significant correlations (P > 0.05).

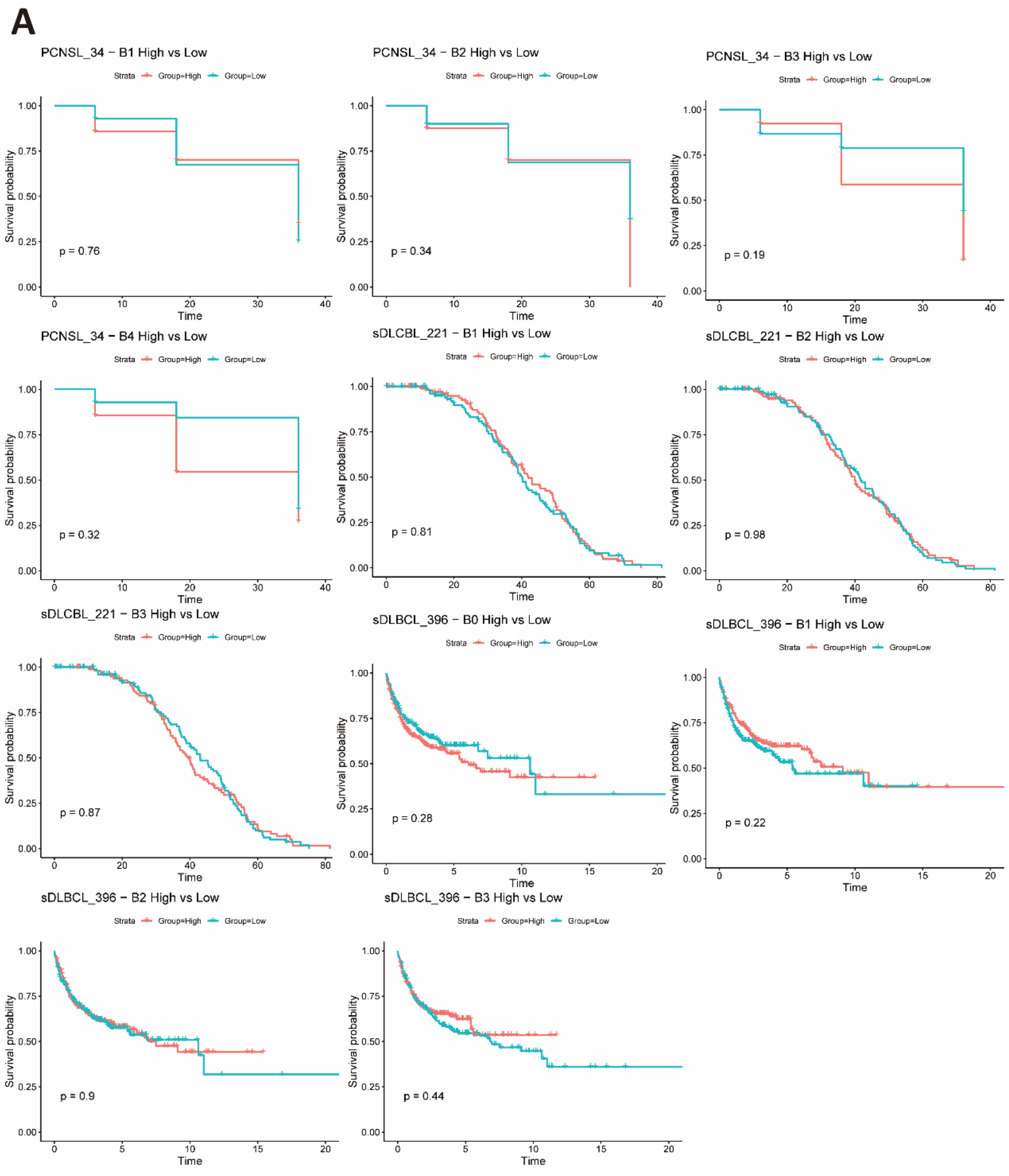

Supplemental Figure S7. Prognostic specificity of B-cell subsets across lymphoma cohorts. A

Kaplan-Meier survival analyses of other B-cell subsets (B1-B4) in PCNSL (N=34), sDLBCL (N=221), and sDLBCL (N=396) cohorts, as well as the B0 subset in the sDLBCL (N=396) cohort. Patients were stratified into High and Low groups based on the median cell fraction. P-values were calculated using the log-rank test. None of these associations reached statistical significance (P > 0.05), highlighting the specific prognostic relevance of B0 in the PCNSL and sDLBCL (N=221) cohorts.

A

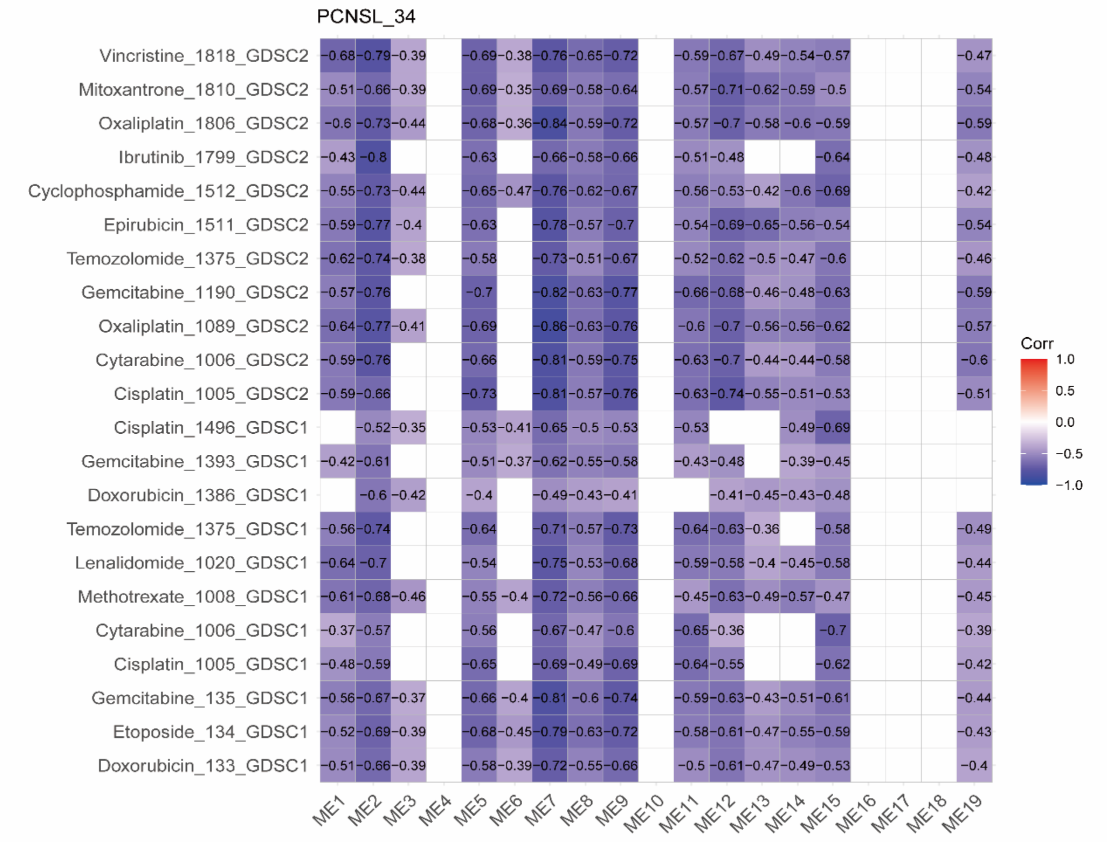

Supplemental Figure S8. Drug-module correlation heatmap in PCNSL. A The heatmap illustrates the correlation between the IC50 values of 22 drugs (representing drug sensitivity) and the scores of 19 gene co-expression modules in PCNSL (N=34). Only statistically significant correlations (P < 0.05) are highlighted with color.

Table S1. Clinical characteristics of the internal study cohort

Sample	Age	Gender	Diagnosis	Cohort	Immunohistochemistry (IHC)	Puncture site	Raw counts	QC_DF counts
p2_PL	68	Female	PCNSL	1	CD20+, CD79a+, CD3 (scattered+), CD5 (scattered+), CD30 (occasional+), BCL6 (80%+), CD10−, MUM1+, PAX5+, Ki-67 ~90%, Cyclin D1 (occasional+), BCL2+, p53+, c-MYC (strong+, ~30%), EBER (ISH)−	Left thalamus	11372	10773
p3_PL	22	Male	PCNSL	1	-	Right frontal lobe	11069	10509
p4_PL	80	Male	PCNSL	1	CD20 (diffuse+), CD79a (diffuse+), CD3 (scattered+), CD5 (scattered+), CD30 (scattered+), BCL6+, CD10+, MUM1 (partial+), PAX5+, Ki-67 ~90%, Cyclin D1 (partial+), BCL2+, p53 (partial+), c-MYC (partial+), EBER (ISH)−, GFAP (glial cells+)	Right frontoparietal region	11192	10617
p7_PL	52	Male	PCNSL	1	CD20+, CD79a+, CD3 (few+), CD5 (few+), CD30−, BCL6+, CD10−, MUM1+, PAX5+, Ki-67 ~80%, Cyclin D1 (scattered+), BCL2+, p53+, c-MYC (~70+%), EBER−, GFAP+, CK−	Right cerebellar hemisphere	11640	11052
p8_PL	55	Female	PCNSL	1	CD20+, CD79a+, CD3−, CD5−, CD30−, BCL6+, CD10−, MUM1+, PAX5+, Ki-67 ~90%, Cyclin D1−, BCL2+, p53+, c-MYC (~60%), EBER (ISH)−, CD23−, CD21−	Left basal ganglia	5678	5389

Table S2. Clinical characteristics of the external cohort

Sample	Age	Gender	Diagnosis	Cohort	Raw counts	QC_DF counts
DLBCL02	47	Female	sDLBCL	2	3641	3426
DLBCL04	55	Male	sDLBCL	2	2271	2126
DLBCL05	73	Male	sDLBCL	2	5580	5220
DLBCL06	51	Female	sDLBCL	2	8531	8015
DLBCL08	10	Female	sDLBCL	2	2292	2108
DLBCL09	64	Male	sDLBCL	2	3362	3161
DLBCL10	60	Male	sDLBCL	2	3097	2912
DLBCL11	24	Male	sDLBCL	2	6277	5912
DLBCL12	48	Male	sDLBCL	2	3949	3696
DLBCL13	73	Male	sDLBCL	2	6154	5769
DLBCL14	35	Female	sDLBCL	2	6595	6199
DLBCL16	59	Female	sDLBCL	2	6764	6390
DLBCL17	76	Female	sDLBCL	2	4496	4203
DLBCL18	52	Male	sDLBCL	2	4222	3790
DLBCL19	40	Male	sDLBCL	2	4989	4697
DLBCL21	66	Male	sDLBCL	2	3731	3475
DLBCL22	59	Male	sDLBCL	2	5392	4819
P124	66	Male	PCNSL	3	4012	3797
P145	50	Male	PCNSL	3	4068	3829
P182	34	Male	PCNSL	3	7476	7058
P201	NA	Male	PCNSL	3	6357	6031
P202	66	Female	PCNSL	3	6726	6265
P203	68	Male	PCNSL	3	9256	8739
P205	60	Male	PCNSL	3	5198	4899
P73	63	Male	PCNSL	3	3905	3700
P73-2	63	Male	PCNSL	3	2896	2746

Table S3. Summary of B cell state counts in samples and subtypes

Sample	Malignant B	Normal B
DLBCL02	8	421
DLBCL04	133	1006
DLBCL05	1698	3090
DLBCL06	57	1463
DLBCL08	567	291
DLBCL09	212	1921
DLBCL10	80	1529
DLBCL11	818	3409
DLBCL12	415	341
DLBCL13	4110	1106
DLBCL14	1919	1821
DLBCL16	135	3517
DLBCL17	775	606
DLBCL18	2335	198
DLBCL19	362	2783
DLBCL21	1295	444
DLBCL22	3416	931
P124	764	2
P145	544	4
P182	4638	5
p2_PL	6466	2
P201	4780	10
P202	3195	18
P203	1500	41
P205	2234	4
p3_PL	10359	26
p4_PL	439	8615
p7_PL	9881	676
P73	1116	17
P73-2	1649	21
p8_PL	5034	3
**Subtype**	**Malignant B**	**Normal B**
B0	23065	7253
B1	17833	10163
B2	15636	11508
B3	8383	3524
B4	6017	1873

Table S4. List of chemotherapeutic agents and targeted drugs included in the drug sensitivity analysis for PCNSL and sDLBCL cohorts

Cohort	Treatment Setting	Drug List
sDLBCL	First-line Therapy	Rituximab, Cyclophosphamide, Doxorubicin, Epirubicin, Vincristine, Etoposide, Prednisone, Polatuzumab vedotin
	Second-line / Relapsed	Rituximab, Cisplatin, Cytarabine, Dexamethasone, Ifosfamide, Carboplatin, Etoposide, Gemcitabine, Methylprednisolone, Cyclophosphamide, Oxaliplatin, Mesna, Mitoxantrone, Lenalidomide, Ibrutinib
PCNSL	Primary Therapy	Methotrexate, Rituximab, Cyclophosphamide, Cytarabine, Thiotepa, Vincristine, Etoposide, Procarbazine, Temozolomide, Prednisone, Carmustine, Lenalidomide
	Relapsed / Refractory	Rituximab, Lenalidomide, Ibrutinib, Zanubrutinib, Orelabrutinib, Temozolomide, Etoposide, Cytarabine, Thiotepa, Liposomal Doxorubicin, Dexamethasone
**Intersection of drugs in the GDSC database**		**Vincristine, Mitoxantrone, Oxaliplatin, Ibrutinib, Cyclophosphamide, Epirubicin, Temozolomide, Gemcitabine, Cytarabine, Cisplatin, Doxorubicin, Lenalidomide, Methotrexate, Etoposide**

Table S5. Topological ranking of hub genes in the PPI network based on degree centrality

Rank	Gene Symbol	Degree (Connectivity)	Description
1	JUN	18	Transcription factor AP-1 subunit Jun
2	FOS	15	Proto-oncogene c-Fos
3	DUSP1	13	Dual specificity protein phosphatase 1
4	FOSB	13	Protein FosB
5	JUNB	12	Transcription factor jun-B
6	ZFP36	12	Tristetraprolin (TTP)
7	NR4A1	12	Nuclear receptor subfamily 4 group A member 1
8	EGR1	10	Early growth response protein 1
9	CD69	9	Early activation antigen CD69
10	PPP1R15A	9	Protein phosphatase 1 regulatory subunit 15A
11	KLF2	9	Krueppel-like factor 2
12	NR4A2	9	Nuclear receptor subfamily 4 group A member 2
13	IER2	9	Immediate early response gene 2 protein
14	KLF6	8	Krueppel-like factor 6
15	PTPRC	7	Receptor-type tyrosine-protein phosphatase C (CD45)
16	TSC22D3	6	TSC22 domain family protein 3 (GILZ)
17	TXNIP	3	Thioredoxin-interacting protein
18	UBC	2	Polyubiquitin-C
19	HLA-B	2	HLA class I histocompatibility antigen, B alpha chain
20	CYTIP	2	Cytohesin-interacting protein
21	HERPUD1	1	Homocysteine-responsive endoplasmic reticulum-resident ubiquitin-like domain member 1
22	CD55	1	Complement decay-accelerating factor
